# Effects of long-lasting social isolation and re-socialization on cognitive performance and brain activity: a longitudinal study in *Octodon degus*

**DOI:** 10.1038/s41598-020-75026-4

**Published:** 2020-10-27

**Authors:** Daniela S. Rivera, Carolina B. Lindsay, Carolina A. Oliva, Juan Francisco Codocedo, Francisco Bozinovic, Nibaldo C. Inestrosa

**Affiliations:** 1 GEMA Center for Genomics, Ecology and Environment, Facultad de Estudios Interdisciplinarios, Universidad Mayor, Santiago, Chile; 2 Center of Aging and Regeneration UC (CARE-UC), Departamento de Biología Celular y Molecular, Facultad de Ciencias Biológicas, Pontificia Universidad Católica de Chile, Santiago, Chile; 3 Center for Applied Ecology and Sustainability (CAPES), Departamento de Ecología, Facultad de Ciencias Biológicas, Pontificia Universidad Católica de Chile, Santiago, Chile; 4 Centro de Excelencia en Biomedicina de Magallanes (CEBIMA), Universidad de Magallanes, Punta Arenas, Chile

**Keywords:** Long-term memory, Short-term memory, Cognitive neuroscience, Stress and resilience, Long-term depression

## Abstract

Social isolation is considered a stressful situation that results in increased physiological reactivity to novel stimuli, altered behaviour, and impaired brain function. Here, we investigated the effects of long-term social isolation on working memory, spatial learning/memory, hippocampal synaptic transmission, and synaptic proteins in the brain of adult female and male *Octodon degus*. The strong similarity between degus and humans in social, metabolic, biochemical, and cognitive aspects, makes it a unique animal model that can be highly applicable for further social, emotional, cognitive, and aging studies. These animals were socially isolated from post-natal and post-weaning until adulthood. We also evaluated if re-socialization would be able to compensate for reactive stress responses in chronically stressed animals. We showed that long-term social isolation impaired the HPA axis negative feedback loop, which can be related to cognitive deficits observed in chronically stressed animals. Notably, re-socialization restored it. In addition, we measured physiological aspects of synaptic transmission, where chronically stressed males showed more efficient transmission but deficient plasticity, as the reverse was true on females. Finally, we analysed synaptic and canonical Wnt signalling proteins in the hypothalamus, hippocampus, and prefrontal cortex, finding both sex- and brain structure-dependent modulation, including transient and permanent changes dependent on stress treatment.

## Introduction

Social stressors are a potent risk factor for the health and well-being across many species. The study of the main stressor types, the importance of timing when stressors occur, and other factors have led to greater understanding of their imperative impact on physiology and behaviour^1^. Social isolation stress (SIS) has a fundamental significance in most social species that depend on parental care, social organization, thermoregulatory huddling, nursing, and predator protection throughout the course of their life-span^2,3^. The effects induced by SIS depend on the species, gender, age, stage of life (early life, adolescence, adulthood or aging), magnitude and duration of the isolation, as well as the perception of the stressor by the individual itself^1,4^.

Studies in rodents have shown that major long-lasting consequences caused by the lack of social interaction have been associated with behavioural abnormalities such as cognitive deficits, including attention disruption, impaired recognition memory, reversal learning, and inability for decision-making^4–7^. In addition, SIS produces several neurobiological changes in the brain, such as deficient neuronal structure and altered brain activity^8–11^. In response to SIS, humans and non-human animals activate a wide array of behavioural, physiological, and neurochemical mechanisms known as the ‘stress response’^1,4,12^. Moreover, sexual dimorphisms can also contribute to the effect of stress responses along life, what could lead to sex-dependent vulnerabilities^13^.

When the ‘stress response’ is triggered, a rise in plasma glucocorticoid (GC) levels (e.g., cortisol or corticosterone), due to the previous activation of the hypothalamic–pituitary–adrenal (HPA) axis, closely follows the initial activation of the sympathetic nervous system^4,12,14^. Indeed, under repetitive stress, the body will generate a GC response that anticipates the next stress episode, therefore having different impacts during juvenile and adult stages^14–16^. In this way, the biological effects of GCs are usually adaptive, becoming essential in the regulation of metabolic, cardiovascular, and immune systems, and in the modulation of learning strategies and memory processes^16–18^. If GC secretion is frequent and prolonged, the allostatic load increases, leading to pathophysiological consequences of cognitive functions^18–22^. Therefore, depending on the circumstances, GCs affect cognitive performance in a context-specific manner that can be either adaptive or pathological^16^.

There are several mechanisms that have evolved to regulate HPA axis activation^12,16,23^. The GC negative feedback response, a process wherein end-products of the stress response inhibit their own release^24^, plays a prominent role in returning homeostasis to the HPA axis following stress exposure^12,25–28^. During early life, maternal deprivation causes a prolonged response to stress beyond stimulus and decreased GC negative feedback^11,29–32^. While in adulthood, the strength of negative feedback system is highly dependent on the stress triggered GC-levels across all stages of life^33^. However, it is the disruption of the mother-newborn relationship, the strongest psychosocial stressor of early life stress that can result in permanent changes, including physical- and emotional-behavioural alterations along adulthood^34–36^. On the other hand, there are some conditions that enhance GC negative feedback efficacy^26,37^. For instance, early handled and/or increased maternal care appear to increase GC receptor expression in rodents, resulting in lower responsiveness of the HPA axis (enhanced negative feedback)^32,37,38^. In this context, several studies in primates and rodents endorse the importance of social interactions to attenuate or even eliminate the HPA activation related to stress responses^39–43^, through a process known as social buffering^14,44^.

Several animal models have been used to investigate SIS and the underlying mechanisms through which SIS exerts its effects on brain function, behaviour and cognition^4,45^. The social rodent species *Octodon degus* (hereafter called degus) has become an increasingly popular animal model to study social-affective biological aspects under stressful conditions^46,47^. This animal lives in average 7 years in captivity, making it an extraordinarily useful rodent model for longitudinal studies^48^. Furthermore, female degus show large oestrous cycle (lasting 17–21 days)^49^, which minimizes the hormonal cycling fluctuations every four days that typically occurs in mice and rats, facilitating the design of long-term studies^50^. In addition, degus exposed to SIS conditions showed a robust stress response along with decreased social motivation, impaired neuronal transmission, and impaired cognition^46,50,51^. However, social-buffering of just one hour per day is able to achieve positive changes and mitigate the effects of social isolation^46,52^. In the present study, we investigated the effects of long-term social isolation on spatial learning memory (hippocampus-dependent), and examined the possibility of reversibility through long lasting re-socialization. We also explored some feasible neural and synaptic mechanisms that could underlie effects under different degrees of SIS, and consider sex variation in these outcomes.

## Materials and methods

### Animals and social isolation protocol

**Figure 1:**
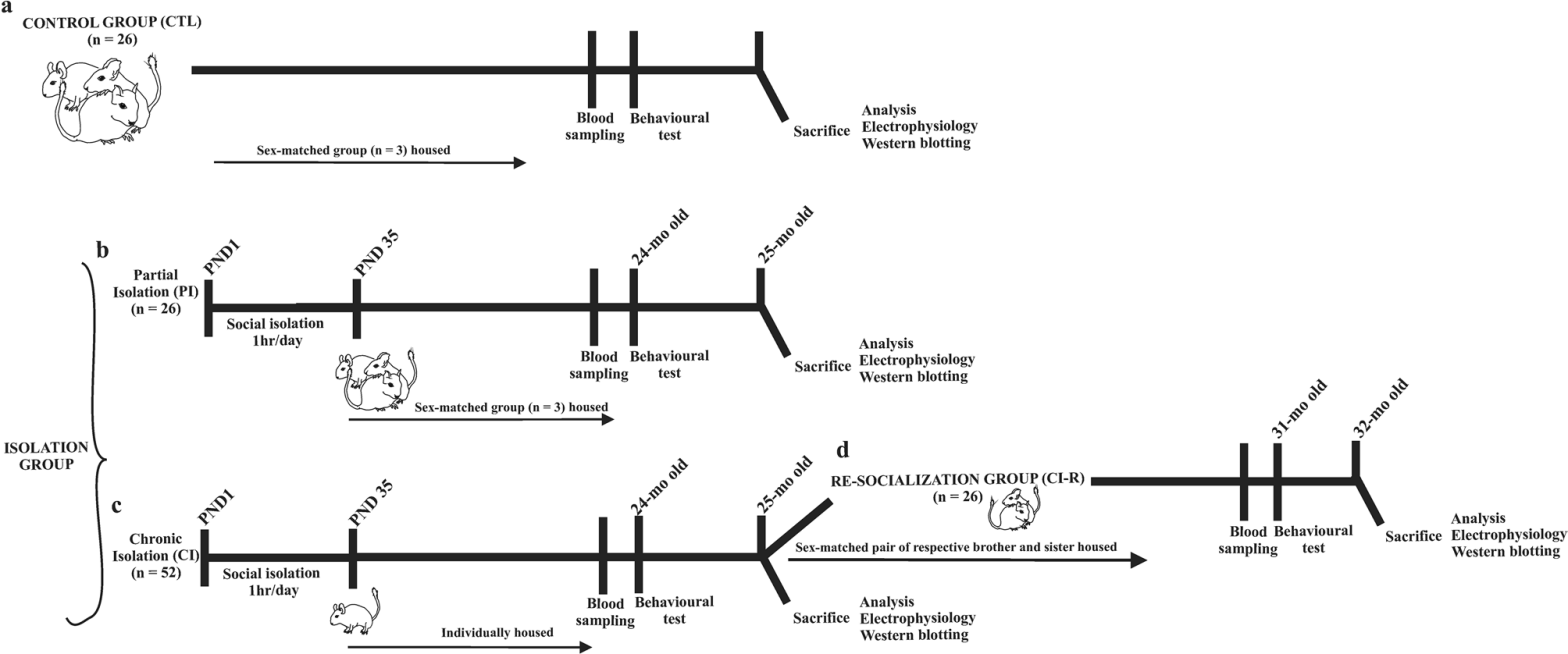
Scheme of experimental design of the stress treatments: (**a**) unstressed control animals (CTL), where litters were left undisturbed with their family. The siblings remained together until PND 90, and thereafter they were raised in sex-matched groups of three siblings from PND 91 until the end of the experiment (n = 13♀ and 13♂). (**b**) Partial isolation group (PI), from PND 1 to PND 35 (day of weaning), the degu pups were removed from their mothers and home cage and were kept individually for one hour daily. After one-hour of separation pups were returned to their family and home cage and left undisturbed until the next day. After PND 35 the whole litter was left undisturbed with their family. The siblings remained together until PND 90, and thereafter they were raised as sex-matched groups of three siblings (one focal degu and two respective brothers/sisters that were not included in our experimental design) from PND 91 until the end of the experiment (n = 13♀ and 13♂). (**c**) Chronic isolation group (CI), from PND 1 to PND 35 (day of weaning), the degu pups were removed from their mothers and home cage and were kept individually for one hour daily. After one-hour of separation pups were returned to their family and home cage and left undisturbed until the next day. From PND 36 until the end of the experiment, female and male degus were individually housed (n = 26♀ and 26♂). (**d**) Re-socialization group (CI-R), after a period of 24-months, half of CI-reared degus were randomly reassigned and housed in sex-matched pairs with CI-reared brothers or sisters (one focal degu and one sibling not included in our experimental design) during a period of 6-month (n = 13♀ and 13♂).

Laboratory pregnant female degus obtained from our colony at the Faculty of Biological Sciences, Pontificia Universidad Catόlica de Chile were kept in pairs and housed in clear acrylic terrariums (length x height x depth: 50 × 35 × 23 cm) with hardwood chip bedding. Each cage contained one nestbox made of clear acrylic (22 × 12 × 15 cm). We checked for litters daily, and the day of birth was defined as postnatal day (PND) 0. To avoid litter differential parental effects within a litter, the whole litter was randomly assigned to one of the following rearing conditions: (i) unstressed controls: the litters were left undisturbed with their family. The siblings remained together until PND 90, and thereafter they were raised as sex-matched groups of three siblings from PND 91 until the end of the experiment (Control group, CTL, Fig. 1a); (ii) Separation stress: from PND 1 to PND 35 (day of weaning), the pups were removed from their mothers and home cage. In the same room, the pups were kept individually in small opaque cages for one hour daily (between 09:00 a.m. and 12:00 p.m. noon). Thus, during separation the pups had acoustic and olfactory but no visual and social contact with their siblings or mother. After one-hour of separation, pups were returned to their family and home cage and left undisturbed until the next day. After PND 35 the whole litter was randomly assigned to one of the following rearing conditions: (ii-a) The litters were left undisturbed with their family. The siblings remained together until PND 90, and thereafter they were raised as sex-matched groups of three siblings (one focal degu and two respective brothers/sisters that were not included in our experimental design) from PND 91 until the end of the experiment when degus reached 25-months old (Partial isolation group, PI, Fig. 1b). (ii-b) From PND 36 until the end of the experiment, male and female degus were individually housed in standard rodent cages, where they had olfactory, acoustic, partial visual, but not physical contact with conspecifics (Chronic isolation group, CI, Fig. 1c). A total of 52 animals: 26 female and 26 male degus (n = 13 per group) were analysed per behavioural test (see above) for CTL and PI condition. For the CI condition a total of 52 animals (26 females and 26 males were analysed). To study the protective role of social buffering after long-term social isolation (25-months), half of CI-reared degus (13 females and 13 males) were housed in sex-matched pairs with their respective CI-reared brothers or sisters (one focal degu and one sibling not included in our experimental design) during a re-socialization period of 6-months, when degus had 31-months old (Re-socialization group, CI-R Fig. 1d).

In our design, we avoided the effect of hormonal fluctuation in 17–21-day regular cycling females, by sampling blood and performing behavioural tests in the diestrous phase of the oestrous cycle. All animals were kept in a ventilated room and exposed to a 12L:12D and ambient temperature (yearly minimum = 13.4 ± 0.2 °C; yearly maximum = 24.9 ± 0.2 °C). Degus were fed a standard rabbit commercial pellet diet (Champion, Santiago, Chile) and ad libitum water. All animal protocols followed the guidelines of the National Institutes of Health (NIH, Baltimore, MD, USA). All procedures were approved by the Bioethical and Biosafety Committee of the Faculty of Biological Sciences of the Pontificia Universidad Católica de Chile (CBB-121-2013). The efforts were made to minimize animal suffering and to reduce the number of animals used.

### Endocrine assays

Cortisol is the predominant glucocorticoid hormone in degus^53^ then, plasma cortisol analysis was performed following previously described protocol^54^. Briefly, three weeks before the behavioural tests (see below) degus were sampled in the afternoon (13:00), so they were undisturbed during more than 4 h before sampling. We sampled three degus per day. Because baseline cortisol (basal CORT) levels start to increase within 3 min of disturbance^55,56^, baseline blood samples (30 µL) were taken within 3 min of entering the room. A second blood sample (15 µL) was taken 30 min after disturbance in order to determine how much degus increased cortisol secretion in response to the stress of handling and blood sampling (stress-induced CORT). Degus were then immediately given an intraperitoneal injection of dexamethasone (DEX) at a dose of 1 mg/kg of body weight, and a third blood sample (30 µL) was taken 90 min later (post-DEX CORT). DEX is a synthetic glucocorticoid and is commonly used to assess negative feedback efficacy, which indicated how well an animal decreases CORT levels after experiencing a stressor^54,57^. It was measured as the percent decrease in cortisol from stress-induced levels to those 90 min after DEX injection: (stress-induced CORT – post-DEX CORT) / (stress-induced CORT) *100^54^.

### Behavioural tests

Degus 25-months old from CTL, PI and CI-reared conditions were subjected to three behavioural trials, as detailed below. Whereas for the CI-R condition, the behavioural tests were performed at the end of the 6-month re-socialization period (i.e., 31-months). To minimize the effects of behavioural experiences on the results, the experiments were conducted in order, from the least to the most intrusive. This order was as follows: i) open field test; ii) the novel object recognition test, and iii) Barnes maze test. Animals were subjected to one test per day (except the Barnes maze test, which require more days). All behavioural tests were performed during the active phase of the animals (between 09:00 to 16:00 h). At the end of each session, animals were returned to their home cages, and the area was wiped clean with a 70% ethanol solution.

#### Open field test

Animals were observed for 5 min in the open field test arena that consisted of a white Plexiglas box (length x height x depth: 100 × 100 × 100 cm). The frequency of total crossings and “central crossings’’ (with a four-paw criterion) were scored^58^. In addition, the percentage of time in corners, in the middle arena, speed, and total length were assessed^58^.

#### Novel object recognition test

The Novel object recognition test is a double test used to evaluate cognition, particularly working memory and attention, but also can be used to test the preference for novelty in rodents^59^. The test arena used an open box (length x height x depth: 63 × 40 × 30 cm) made of white Plexiglas. For this test, we followed the object recognition protocol previously used in degus^58^. Briefly, animals were exposed to a 10 min familiarization session and then tested in two consecutive 5 min sessions, with a 1 h inter-session interval. For Session 1 (Familiarization): two different objects (“Object A” and “Object B”) were placed in the corners of the home cage and the animal was allowed to freely explore them for 10 min. Following this period, objects were removed from the cage and wiped with a 70% ethanol solution and the test animal was returned to its home cage for 1 h. For Session 2 (“Novel location recognition” or NLR): one of the familiar objects (Object B) was moved to an adjacent unoccupied corner. The test animal was then free to interact with the objects during 5 min. Following this period, objects were removed from the cage and wiped with a 70% ethanol solution and the test animal was returned to its home cage for 1 h. For Session 3 (“Novel Object Recognition” or NOR): the familiar Object B was replaced by a different, but similar, object. We recorded the familiarization and testing times, and the time spent exploring each object. “Exploration” time was defined as approaching to within 1–3 cm of the object. To quantify NLR and NOR, a Recognition Index (RI) was calculated as the time spent with object B divided by the sum of the time spent with object A and object B. A normal and unstressed rodent would tend to explore the novel object more than the familiar one. Then, a RI above 0.5 indicates greater investigation of the novel location or object.

#### Barnes Maze test

This test has a strong hippocampal-dependent spatial component^60^, leading us to evaluate spatial navigation, learning and memory^61^. The Barnes maze consisted of a circular elevated platform made of white Plexiglas 160 cm in diameter and surrounded with 45 cm high wall. Eighteen circular holes (8 cm in diameter), were bored through the platform equidistant from each other (16 cm), and 5.5 cm from the outer edge. All holes except the target one were blocked. A plastic escape box (length x height x depth: 31 × 13 × 16 cm) was positioned under the escape hole. Accurate performance requires subjects to learn and remember the location of the escape hole, therefore spatial cues (combination of different colours and shapes: a yellow star, a red square, and a green apple) were placed on the wall of the maze^62^. Briefly, the procedure was divided into three phases: habituation, training, and test phases, and was performed as previously described^58,63^. Session 1 (Habituation): began when the focal degu was placed into the escape box for 2 min. Subsequently, the animal was placed near the escape hole and left for 1 min to give it the chance to escape. If the animal did not enter the escape box, it was gently picked up and pushed through the target hole inside the escape box and left there for 2 min. Finally, the animal was placed at the centre of the maze and left there for 4 min to explore the platform and enter the escape box. In case the animal did not enter in the escape box, it was put into the escape box as mentioned above and left there for another 2 min. Session 2 (Training): 2 days after Session 1, we trained each animal for 7 days. Session 3 (Test phase): 7 days after Session 2, we exposed the test animals to a memory-retrieval session. Both training and test phase consisted of four consecutive 4 min trials, separated by a 5 min resting phase in the animal home cage. At the beginning of each trial, the animal was confined for 30 s in a start box in the centre of the maze. If the animal did not enter the escape box within the allotted time, it was manually picked up and placed in the escape box, where it remained undisturbed for 2 min. The surfaces of the maze platform were cleaned with 70% ethanol between trials. To control for locomotor differences between groups, we measured the speed and the distance (in meters) covered from the initiation of exploration of the escape hole to entrance into the escape box. We registered the “latency to the first visit of escape hole”, the “reference memory errors” (every first visit of a non-escape hole in each trial) and “working memory errors” (repeated visits to the same non-escape hole in the same trial). The ‘search strategies’ used during retrieval trials were categorized into three groups: random, serial, and spatial as previously described^64,65^. Briefly, searches were classified as ‘random’ when localized searches of the escape hole were interrupted by central crosses or when no systematic search pattern was discernible. ‘Serial searches’ were defined as searches in a clockwise or counter clockwise direction, and ‘spatial searches’ were defined as searches following a direct path to the escape hole.

In every case a digital video camera (Microsoft® Webcam LifeCam Studio Full HD) was mounted above the arena test and the performance of each animal was monitored with image tracking software (HVS Image, Hampton, UK).

### Brain slice preparation for electrophysiological measurements

At the end of the behavioural tests, 32-months old degus were euthanized by decapitation after isoflurane deep anaesthesia. The brain was quickly removed and placed in cold artificial cerebrospinal solution (ACSF)-modified (sucrose replaces part of sodium), oxygenated with 95% O_2_/5% CO_2_, composed of the following (in mM): 85 NaCl, 75 sucrose, 3 KCl, 1.25 NaH_2_PO_4_, 25 NaHCO_3_, 10 dextrose, 3.5 MgSO_4_, 0.5 CaCl_2_, 3 sodium pyruvate, 0.5 sodium L-ascorbate and 3 myo-inositol (305 mOsm, pH 7.4). We used this solution to cut coronal sections of 350 µm with a vibratome, and the slices were kept for recovery during 1 h in the same solution but at 36 °C. After that, we changed the solution to an oxygenated ‘recording solution’, composed of (in mM): 126 NaCl, 3.5 KCl, 1.25 NaH_2_PO_4_, 25 NaHCO_3_, 10 dextrose, 1 MgSO_4_, 2 CaCl_2_, 3 sodium pyruvate, 0.5 sodium L-ascorbate and 3 myo-inositol (305 mOsm, pH 7.4) at room temperature (22 °C) and slices were kept there until recording. Each slice was placed on a recording chamber allocated on an upright infrared-differential interference contrast (IR-DIC) fluorescence microscope (Eclipse FNI, Nikon). The hippocampal circuit was visualized with a 40 × water objective and a light-sensitive camera (TOPICA CCD Camera). We used a bipolar concentric electrode (World Precision Instruments, Sarasota, FL, United States) to stimulate the Schaffer collaterals between CA3 and CA1, connected to an ISO-Flex stimulus generator (A.M.P.I., Jerusalem, Israel). To record the evoked field excitatory postsynaptic potentials (fEPSPs), we placed a borosilicate glass electrode (World Precision Instruments, United States) of 0.5–1 MΩ pulled on a P-97 Flaming/Brown Micropipette Puller (Sutter Instruments, United States) and filled with recording solution, on the *stratum radiatum* of CA1. The signals were recorded using a MultiClamp 700B amplifier (Axon CNS, Molecular Devices LLC, United States), and digitally sampled at 30 kHz using a Digidata-1440A interface (Axon CNS, Molecular Devices). All the analyses were done offline using pClamp 10.3 (Molecular Devices LLC, United States). We applied several protocols to characterize the effect of social isolation treatments on this neural circuit. To establish the relationship between the stimulus intensity and the extent of evoked response, we obtained the input–output (I-O) curve by plotting the fEPSP slope measurements against increasing levels of current intensity. We used the amount of current that evoked 60% of the maximum slope value to perform other protocols in the same slice. To evaluate synaptic plasticity, long-term plasticity (LTP) experiments were performed. Two pulses (R1 and R2) separated by 50 ms each, were applied every 15 s with the stimulus generator. The fEPSP slope of the first pulse (R1) was averaged during 15 to 20 min to obtain a stable basal signal. Then, a theta burst stimulation (TBS, 5 bursts at 100 Hz every 20 s) was applied. After TBS, the same two pulses separated by 50 ms were applied for at least 60 min, counted as post-TBS period. The fEPSP slope measurements (R1) obtained during the post-TBS period, were compared to the averaged slope measurements obtained before TBS to determine the degree of potentiation. The plot represents the relative value of fEPSP slope along time and between groups.

### Western blot analysis

The hippocampus, prefrontal cortex and hypothalamus of CTL, PI, CI, and CI-R degus (n = 3 respectively) were dissected on ice and immediately frozen at − 150 °C to be processed as previously described^58^.

Briefly, the hippocampal tissues were homogenized in RIPA buffer (50 mM Tris–Cl, pH 7.5, 150 mM NaCl, 1% NP-40, 0.5% sodium deoxycholate, and 1% sodium dodecyl sulphate [SDS]) supplemented with a protease inhibitor cocktail (P8340, Sigma-Aldrich, Germany) and phosphatase inhibitors (50 mM NaF, 1 mM Na_3_VO_4_, and 30 μM Na_4_P_2_O_7_) using a Potter homogenizer; then, the samples were passed sequentially through different caliber syringes. Protein samples were centrifuged twice at 20.817 × *g* at 4 °C for 15 min.

The protein concentration was determined using a Bicinchoninic Acid Protein (BCA) Assay Kit (Thermo Scientific). 20 and 60 μg of the protein samples were separated by 10% SDS-PAGE and transferred to a polyvinylidene difluoride membrane. The membranes were incubated with primary antibodies: postsynaptic density 95 [PSD-95] and synaptophysin [SYP] (Abcam, Cambridge, UK); N-Methyl-D-aspartate receptor (NMDAR) subunit 2B [NR2B] (Sigma-Aldrich, St. Louis, USA); total β-catenin and total GSK3β (Santa Cruz Biotechnology, Inc., Santa Cruz, CA, USA); pS9-GSK3β (Cell Signalling Technology), and pY216-GSK3β (BD Transduction Laboratories; Franklin Lakes, NJ, USA). Samples were then washed with PBS-Tween 0.1% or 0.05% and incubated with secondary antibodies: anti-mouse, anti-rabbit and anti-goat IgG peroxidase-conjugated antibodies (Abcam). Later, the membranes were developed using an ECL kit (Biological Industries, Israel). To analyse the results, all target protein signals were normalized against the loading control (β-actin from Sigma-Aldrich or GAPDH from Santa Cruz Biotechnology) and the signal of phosphorylated proteins were also normalized against the total protein levels. In the main text, we will refer to the ratio pS9-GSK3β/total GSK3β levels as “inhibited” GSK3β levels (or pS9-GSK3β ratio) while to the ratio pY216- GSK3β/total GSK3β levels as “activated” GSK3β levels (or pY216- GSK3β ratio).

### Statistical evaluation

Prior to conducting univariate analyses (i.e., ANOVA), we explored the data by combining some measures via Principal Component Analysis (PCA). The PCAs were conducted on measures recorded from different behavioural tasks to distinguish among variables which assess qualitatively different aspects of behaviour. Our PCAs used the correlation matrix because our variables were on different scales and this approach standardizes the data^66^. We used the first and second principal components (PC1 and PC2) because the PC1 explained most of the variance and the PC2 explained most of the remaining variation (both of them explained more than 50% of the variation). These PCAs allowed us to determine whether there were sex differences in the relationships between cognitive performance and degree of social isolation. Then, for spatial memory analyses, we used RI information of NLR/NOR. For learning performance, the PCA included information of latency to the first visit of the escape hole and the reference and working memory errors to find the escape hole. We conducted two PCAs: one for female degus and one for male degus across each group of behavioural tasks. To assess statistical significance of group separation, we performed a one-way PERMANOVA with 9999 permutations, which permute the distance matrix (Euclidian method).

For univariate analyses, we used two-way ANOVA to determine the effects of stress treatments, sex, and a stress treatment by sex interactive effect on behavioural tasks. For the endocrine stress responses analysis, we collapsed the cortisol measures of female and male degus as we did not find significant differences between sexes. Therefore, cortisol measurements were examined using one-way ANOVA. In addition, due basal CORT, stress-induced CORT, and negative feedback are regulated independently, we analysed each CORT variable separately^67,68^. For the western blot analysis, we used one-way ANOVA to analyse the effect of stress treatment groups in both female and male degus. For the NOR/NLR test, the RI was analysed using two-way ANOVA. Similarly, to analyse training data of the Barnes maze test, we performed two-way ANOVA with one repeated measure (i.e., time). When appropriate, Fisher’s LSD post-hoc comparisons were performed to examine individual main effects of stress treatments and sex^69^. For electrophysiology analyses, we used n = 3 degus per experimental condition and per sex. At least three brain slices from each animal were considered replicates and were averaged together. Electrophysiological data to measure Input–Output curves were analysed via two-way repeated measures ANOVAs to determine the effect of sex, stimulus and the interaction between two factors. The LTP curves were analysed to determine the effect of sex, time post-TBS and the interaction between both factors. In both cases Bonferroni post hoc test was used when applicable. The assumptions of normally distributed data and homogeneous variances were confirmed using Shapiro–Wilk and Levene's tests, respectively. Statistical analyses were performed using the Statistica (StatSoft, Tulsa, OK) software package. Differences were considered statistically significant at *p* < 0.05.

## Results

### Under long-term chronic social isolation stress degus had weaker cortisol negative feedback, whereas, under re-socialization it was enhanced

#### Baseline cortisol

**Figure 2:**
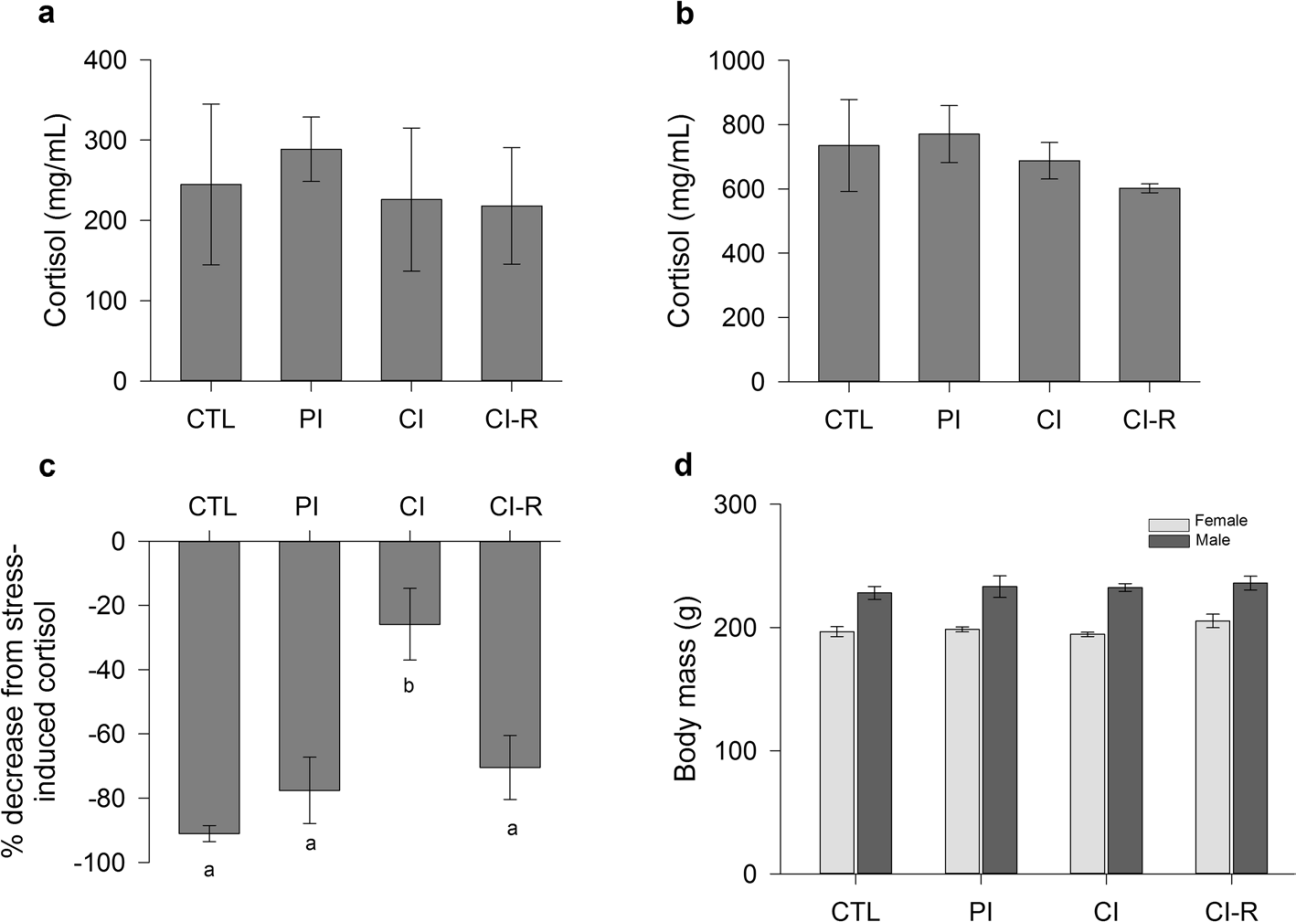
Mean ± SEM (**a**) baseline and (**b**) stress-induced cortisol levels, (**c**) negative feedback efficacy, and (**d**) body mass for Control (CTL), Partial Isolation (PI), Chronic Isolation (CI), and Re-socialization (CI-R) treatment groups (n = 6 per group). Note that a larger decrease indicates stronger negative feedback. Negative feedback efficacy was measured as the percent decrease from stress-induced cortisol levels to those 90 min after dexamethasone injection.

There was no significant differences in baseline cortisol levels across the stress treatments [one-way ANOVA, F_(3,20)_ = 0.16, *p* = 0.92; Fig. 2a].

#### Stress-induced cortisol

There was no significant effect on stress-induced cortisol levels across the stress treatments [one-way ANOVA, F_(3,20)_ = 0.67, *p* = 0.58; Fig. 2b].

#### Negative feedback

Negative feedback significantly differed across stress treatments [one-way ANOVA, F_(3,20)_ = 9.47, *p* < 0.01]. Comparisons between groups revealed that the CI group had significantly weaker negative feedback that the CTL group. Interestingly, CI-R animals showed significantly stronger negative feedback compared to CTL and PI groups (Fig. 2c).

#### Body mass

Body mass was not affected by the stress treatment (*p* = 0.36), but was significantly higher in males than females [Fig. 2d: two-way ANOVA, F_(1,40)_ = 91.96, *p* < 0.01]. No significant interaction was detected between the stress treatment and sex factors (*p* = 0.77).

### Exploratory multivariate analysis

**Figure 3:**
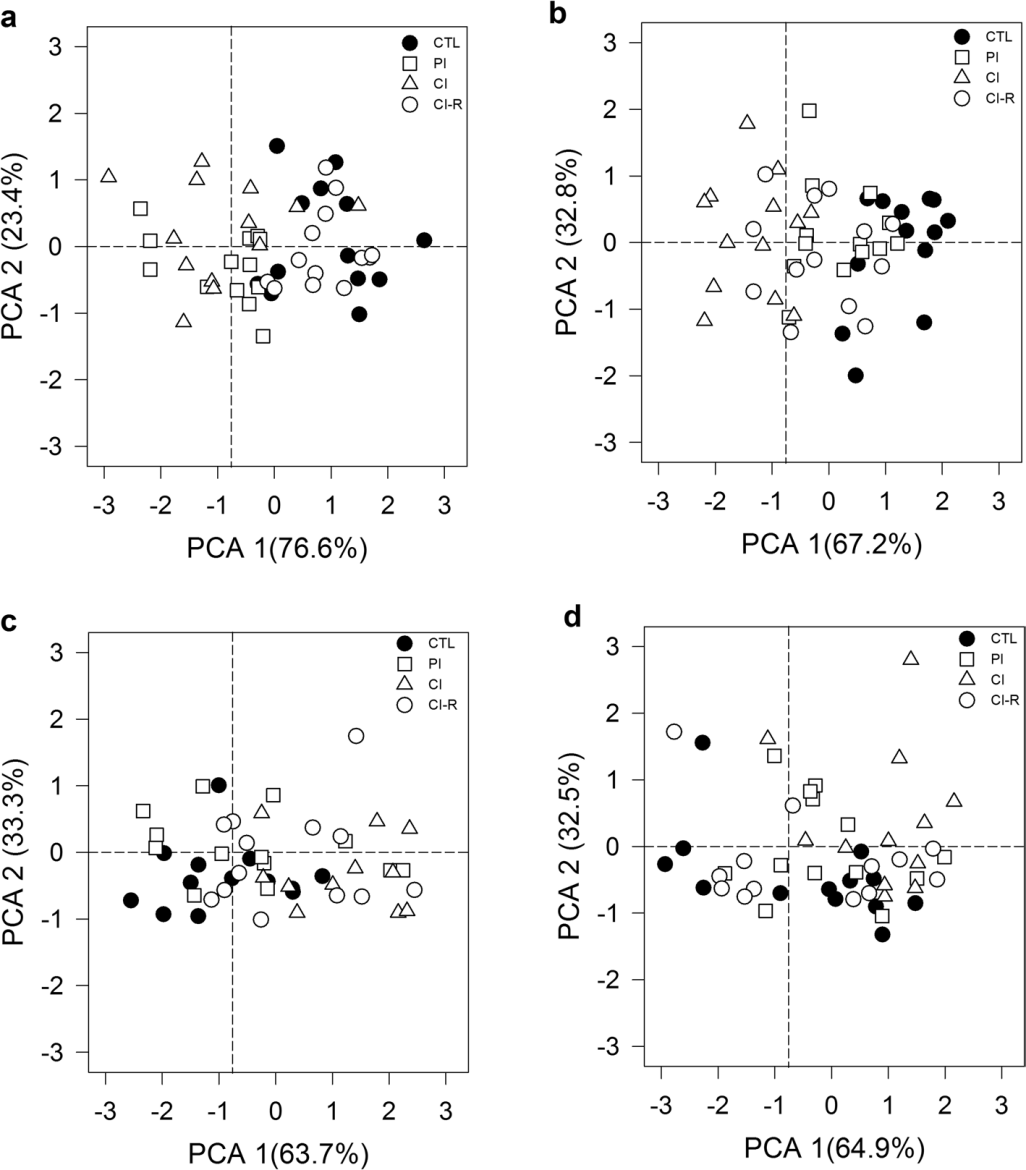
Principal component analysis (PCA) graph of spatial memory (**a**,**b**) and learning performance data (**c**,**d**) across the stress treatments for female (n = 13 per group) (**a**,**c**) and male (n = 13 per group) (**b**,**d**) degus. Each data point represents one degu, and symbols represent each treatment (black circles: Control, CTL group; white squares: Partial Isolation, PI group; white triangles: Chronic Isolation, CI group; white circles: Re-socialization, CI-R group).

Visualization of sample relationships for the spatial memory via PCA analyses showed a clear and significant pattern between stress treatments across female (Fig. 3a) and male individuals (Fig. 3b). Likewise, One-way PERMANOVA analyses confirmed that female CI degus formed a separate group from the CTL and CI-R groups (F = 10.37, *p* < 0.01). For males, CI degus were different from all the three groups (F = 10.91, *p* < 0.01; Fig. 3a), whereas CI-R males formed a separate group more similar to the PI group (Fig. 3b). Importantly, these results reflect a strong pattern on the effect of long-term chronic isolation stress in spatial memory for both female and male degus.

Principal Component Analysis (PCA) for the learning performance did not reveal any trends across the stress treatment for female degus (Fig. 3c; F = 1.81; *p* = 0.12). Whereas for male degus, one-way PERMANOVA confirmed a clear and significant pattern between stress treatments (Fig. 3d; F = 4.21; *p* < 0.01), with CI males being statistically different from CTL and CI-R male degus. This suggest that long-term chronic social isolation stress affects to a great extent the learning performance of males rather than female degus.

### Impairment of cognitive performance by long-term chronic social isolation stress and its recovery by re-socialization

To evaluate the general state of animals, we performed the open field test. In this context, we measured the time that the animal spent in the central zone of the arena, the number of central crossings, the speed, and the total distance travelled. All of them were not affected by stress treatment, by sex, or by the interaction between the two factors, suggesting that general behaviour is not affected by SIS (See Fig. S1a–d in Supplementary Information, SI).

**Figure 4:**
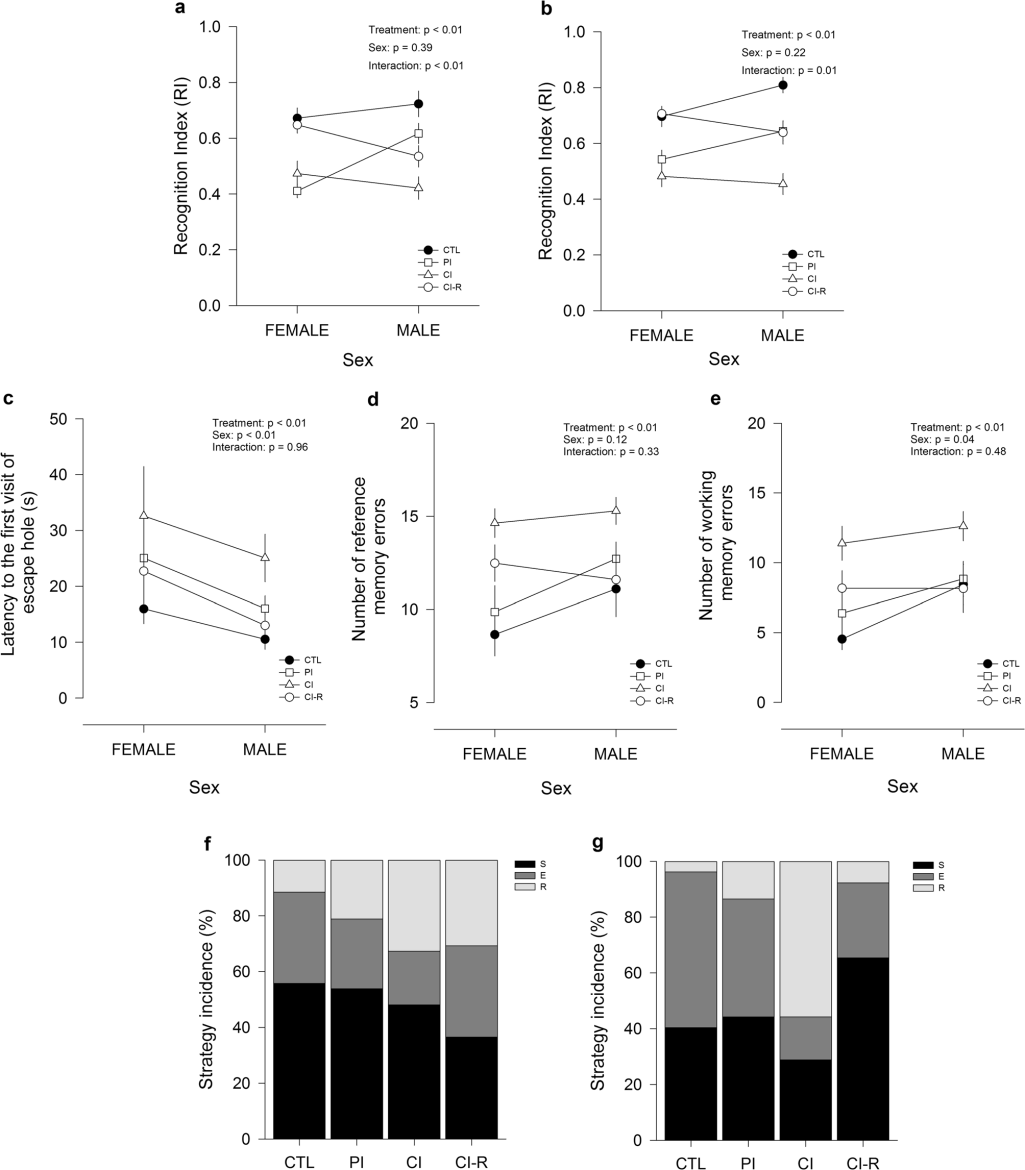
Effect of long-term chronic social isolation stress in Novel Local Recognition/Novel Object Recognition (NLR/NOR) and Barnes maze tests: (**a**) Recognition Index (RI) for NLR (**b**) RI for NOR (**c**) latency to first visit of the escape hole across the test phase of the Barnes maze test (**d**) reference memory errors across the test phase of the Barnes maze test (**e**) working memory errors across the test phase of the Barnes maze test (**f**) search strategies used for female and (**g**) male degus during the test phase of Barnes maze test: S: serial strategy; E: spatial strategy; R: random strategy. Control (CTL), Partial Isolation (PI), Chronic Isolation (CI), and Re-socialization (CI-R) treatment groups. Data were analysed statistically using two-way ANOVA followed by Fisher’s LSD post hoc test. The statistical effect of stress treatment, sex, and the interaction between the two factors are indicated in the top of the figure. Each symbol corresponds to data from a single-sex stress treatment group, represented as the mean ± SEM (n = 13).

In order, to evaluate the impact of long-term chronic social isolation on recognition memory in female and male degus, we evaluated the exploratory motivation of degus to interact with a novel object in the NLR/NOR test. The two-way ANOVA analyses on the RI showed significant effect of stress treatment [RI for NLR: F_(3,96)_ = 16.26, *p* < 0.01; RI for NOR: F_(3,96)_ = 24.97, *p* < 0.01]. These differences were independent of sex (*p* = 0.38 and *p* = 0.22, respectively), but a significant effect of the interaction between the two factors was recorded for NLR and NOR sessions [F_(3,96)_ = 6.05, *p* < 0.01; and F_(3,96)_ = 3.47, *p* = 0.01; Fig. 4a,b, respectively]. Further analysis for session 1, showed that CI degus had lower RI during NLR if compared with CTL and CI-R groups. During NOR session, CI group had the lowest value compared to the other groups. Interestingly, these results showed that degus under long-term chronic social isolation present impairment in working memory, and more importantly, the re-socialization process may help recover the memory impairments and the predilection for novel experiences in both sexes. The time of interaction with a novel object and familiar object data are shown in SI.

To examine the effect of long-term chronic social isolation on spatial learning and memory processes, all animals performed the Barnes maze test. Data related with latency to find the escape hole, the number of reference and working memory errors, and the navigation strategy used during the training phase are shown in SI. During the test phase, the time taken to find the escape hole, a measurement of long-term memory, was significantly affected by stress treatment [F_(3,96)_ = 4.91, *p* < 0.01], sex [F_(1,96)_ = 7.24, *p* < 0.01], but was not affected by the interaction between the two factors (*p* = 0.96; Fig. 4c). Additional comparisons between groups showed that female degus take more time to find the escape hole than males. Within females, no changes were found across the four stress treatments, whereas within males, CI degus required about twice the time to find the escape hole compared with CTL, PI, and CI-R degus. We also measured the speed and total distance travelled; in the case of speed we found that there was no effect of stress treatment (*p* = 0.43), there was a significant sex effect [F_(1,96)_ = 4.57, *p* = 0.03], but was not affected by the interaction between treatment and sex (*p* = 0.79; Fig. S4a in SI). Further analysis indicated that male degus moved significant faster than females. Instead, for the total distance travelled we found a significant effect of stress treatment [F_(3,96)_ = 7.6; *p* < 0.01], but no significant effect of sex (*p* = 0.10) or interaction between the two factors (*p* = 0.26; Fig. S4b in SI). Subsequent analysis indicated that CI degus travelled further than all other groups.

In the case of reference memory errors, we observed a significant effect of stress treatment [two-way ANOVA; F_(3,96)_ = 7.19, *p* < 0.01], however, these results were not dependent on sex (*p* = 0.11) nor on the interaction between both factors (*p* = 0.33; Fig. 4d). Subsequent analysis indicated that CI degus made more reference memory errors than all other groups. Regarding the working memory errors to find the escape hole, our results showed a significant effect of stress treatment [F_(3,96)_ = 6,76, *p* < 0.01], sex [F_(1,96)_ = 4.24, *p* = 0.04], but was not affected by the interaction between the two factors (*p* = 0.48; Fig. 4e). Further analysis indicated that CI and CI-R female degus made more working memory errors compared with CTL female degus. Whereas in male degus we did not observe differences across treatments despite a trend for CI males to make more reference memory errors. Taken together, these results suggest a sex-associated stress- response on spatial learning and memory during test phase.

Furthermore, when we compared the strategy taken by degus to find the escape hole, we observed that CI female degus mostly used a serial- and random-search strategies (48 and 33% respectively), compared to a serial- and spatial-search strategies (56 and 33%, respectively) observed in CTL females (Fig. 4f). Whereas CI-R female degus applied all three strategies homogeneously (serial: 36%; spatial: 33%; random: 31% Fig. 4f). In contrast, CI male degus demonstrated a random- and serial-search strategy pattern (56 and 29%, respectively) compared to CTL male degus, which navigated by mostly using spatial- and serial-search strategies (56% and 40%, respectively; Fig. 4g). Instead, CI-R males used serial- and spatial-search strategies (65 and 27%, respectively; Fig. 4g). These results indicated that, during the test phase of Barnes maze, both CTL female and male degus preferably applied serial- and spatial-strategies in opposite proportions, and that these preferences change by effect of long-term social isolation stress (CI group) toward a random- and serial strategies. Interestingly, CI-R females showed an intermediate mixture of strategies and CI-R males behave similar than CTL, showing that resocialization is effective in recovering spatial learning and memory.

### Effects of long-term social isolation stress on synaptic activity properties in brain slices of degus

**Figure 5:**
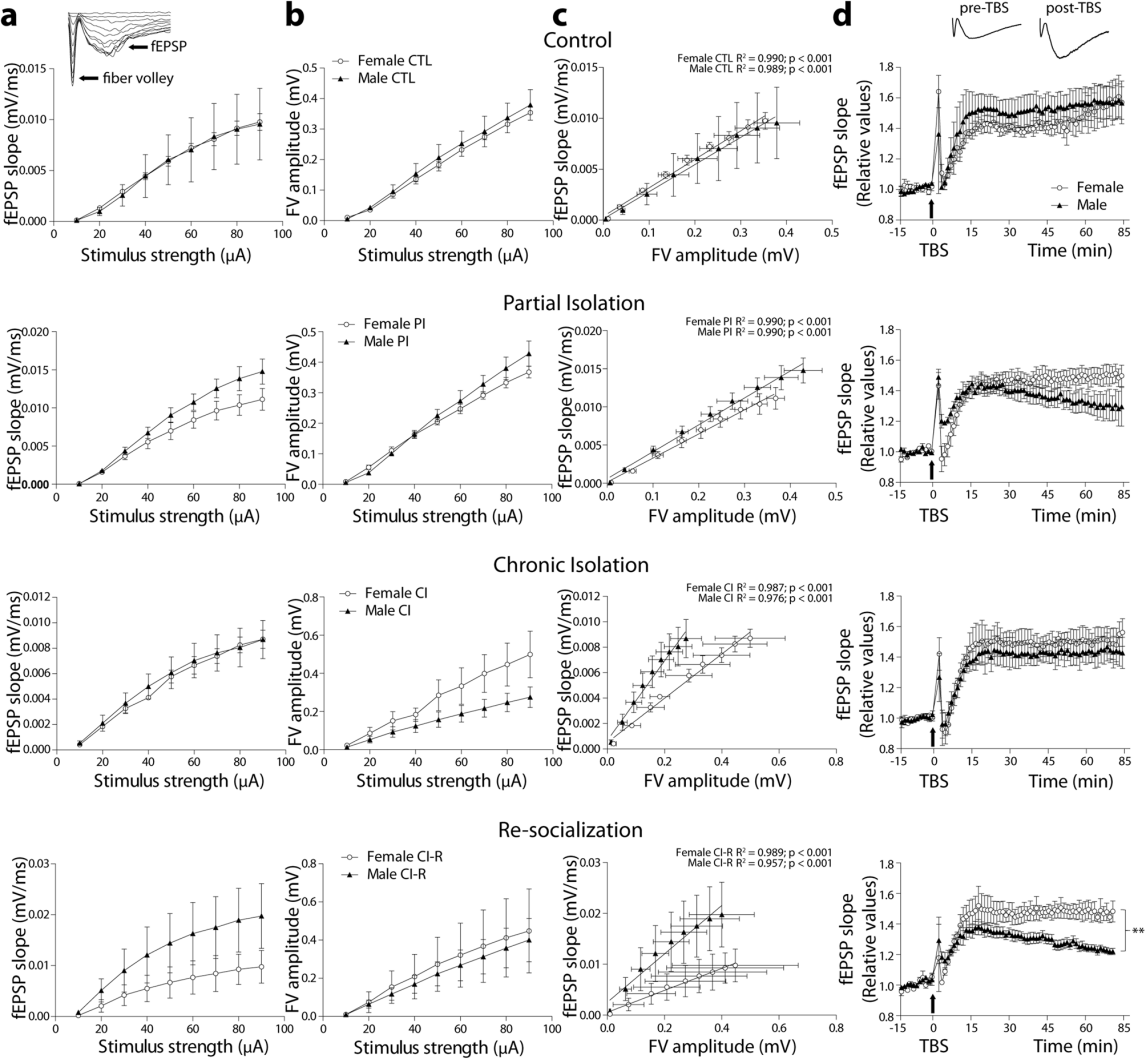
Electrophysiological measurements reveal the modulation of synaptic mechanisms as a consequence of social isolation stressors. (**a**,**b**) Comparison between females (circles) and males (triangles) of input–output activity obtained at different stimulus strengths, and plotted as field excitatory postsynaptic potential (fEPSP) slope (**a**), or fiber volley (FV) amplitude (**b**). The inset showed an example of evoked potential at different increasing stimulus. The correlation of both parameters (right column) was adjusted with linear regression, and the parameters are shown at the top of each graph (**c**) Differences between regressions were evaluated with ANCOVA, showing that basal synaptic transmission in CI males becomes more efficient, and this change persists into CI-R. (**d**) Synaptic plasticity measured as LTP of females and males in each experimental group. The inset showed an example of evoked potential before and after 60 min of TBS. Arrows in graphs indicated the application of theta burst stimulation (TBS). Only CI-R females and males were significantly different. Differences were evaluated by two-way repeated measures ANOVA with Bonferroni post hoc tests, **p < 0.01. (n = 3 degus per experimental condition and per sex).

To elucidate the effect that different social isolation stress treatments have in synaptic transmission and plasticity mechanisms, we performed electrophysiological experiments in brain slices obtained from female and male degus that were under four different SIS conditions (Fig. 5). We stimulated the collateral axons at CA3 and recorded the evoked fEPSPs at the level of the CA1. To evaluate the capacity of the hippocampal circuit to increase the evoked response by increasing the intensity of stimulus, we built an input–output curve by applying increasing levels of current intensities (see Fig. 5a, inset). First, we plot the measurements of the slopes from the resultant fEPSPs (Fig. 5a). The plot of fEPSP slope against stimulus amplitude shows non-significant differences between female and male curves in CTL and CI groups (Fig. 5a; slope at maximum intensity: CTL: 0.0097 ± 0.0005 mV/ms, *p* = 0.75; CI: 0.0087 ± 0.0007 mV/ms, *p* = 0.53). But significant differences in PI (Fig. 5a; slope at maximum current intensity: PI female: 0.0111 ± 0.0014 mV/ms, male: 0.0148 ± 0.0016 mV/ms; two-way repeated measures ANOVA, PI: interaction: F_(8,36)_ = 0.93, *p* = 0.503; sex: F_(1,36)_ = 14.51, *p* < 0.01; stimulus: F_(8,36)_ = 41.62, *p* < 0.01) and CI-R groups (Fig. 5a; slope at maximum current intensity: CI-R female: 0.0098 ± 0.0032 mV/ms, male: 0.0198 ± 0.0064 mV/ms; two-way repeated measures ANOVA: CI-R: interaction: F_(8,36)_ = 0.27, *p* = 0.971; sex: F_(1,36)_ = 10.28, *p* < 0.01; stimulus: F_(8,36)_ = 3.43, *p* < 0.01). We also compared the fEPSPs slopes within each sex, where treatments did not significantly affect basal synaptic activity in females (Fig. S6a in SI; females: *p* = 0.622), but they did it at males (Fig. S6 in SI; males: F_(24,72)_ = 0.38, *p* = 0.995; treatment: F_(3,72)_ = 13.64, *p* < 0.01; stimulus: F_(8,72)_ = 10.49, *p* < 0.01).

On the other hand, we built an input–output curve with the fiber volley (FV) amplitude, which indicates the number of axons recruited during increasing stimulation (Fig. 5b). The plot of FV against stimulus amplitude shows (no differences among female and male curves on CTL, PI and CI-R (Fig. 5b; FV amplitude at maximum current intensity: CTL: 0.362 ± 0.009 mV, *p* = 0.244; PI: 0.398 ± 0.024 mV, *p* = 0.06; CI-R: 0.419 ± 0.094 mV, *p* = 0.43), but they do it at CI group (Fig. 5b; FV amplitude at maximum current intensity: CI female: 0.499 ± 0.122 mV, male: 0.275 ± 0.054 mV; two-way repeated measures ANOVA, CI: interaction: F_(8,36)_ = 0.77, *p* < 0.628; sex: F_(1,36)_ = 15.26, *p* < 0.01; stimulus: F_(8,36)_ = 8.28, *p* < 0.01). Additionally, we compared the FV amplitudes within each sex and we found no differences in females (Fig. S6a in SI; *p* = 0.052) or males (Fig. S6b in SI; *p* = 0.057), demonstrating that SIS treatments did not affect the axonal recruitment during basal synaptic transmission.

In order to test the correlation strength between FV amplitude and fEPSP slope, which would indicate the efficiency of transmission per recruited axon, we plotted the correlation between them and adjusted a linear regression. Then, we compared these correlations within each sex and stress treatment (Fig. 5c). We found a significant positive correlation between FV amplitude and fEPSP slopes in both females and males across all stress treatment groups (See data in plot and details in SI). To determine whether the regressions significantly differed between females and males, we performed ANCOVAs. The results showed that between CTL female and male regressions, there is a significant difference at the intercepts (ANCOVA: F_(1,15)_ = 8.23, *p* = 0.01) but not the slopes (*p* = 0.404). Similarly, between PI females and males, the regressions are significantly different at the intercepts (ANCOVA: F_(1,15)_ = 21.58, *p* < 0.01) and not the slopes (*p* = 0.072). Within the CI group, female and male regressions are significantly different at both intercepts (ANCOVA: F_(1,15)_ = 32.13, *p* < 0.01) and slopes (F_(1,14)_ = 54.48, *p* < 0.01). In the same way, the female and male regressions of the CI-R group showed significant differences at the level of the intercept (ANCOVA: F_(1,15)_ = 59.71, *p* < 0.01) and slopes (F_(1,14)_ = 48.97, *p* < 0.01). We also compared the stress treatments within each sex, and showed in the SI (Fig. S6c,d in SI). These results indicated that as a consequence of stress treatment, females and males displayed different synaptic mechanisms. Indeed, males had more effective basal synaptic transmission, likely through their ability to better modulate the strength of individual axon synaptic transmission in stress treatments.

Next, we evaluated whether stress treatment affected LTP in degus. We used a theta burst stimulation (TBS) to induce LTP, which is the best stimulus to activate the hippocampal memory circuits by mimicking the frequency of hippocampal theta rhythm^70^. A stable recording was obtained for at least 15 min and then a TBS protocol was applied to induce LTP. The recording after TBS for at least one hour was compared to the basal time before TBS and was shown as relative change (Fig. 5d, inset). The plots show that degus can generate significant magnitude of LTP in every condition. We plotted female and male curves for each experimental group and compared the amount of LTP (Average of the last 15 min: CTL female = 56.6 ± 13.1%, CTL male = 57.1 ± 10.8%; PI female = 48.8 ± 6.2%, PI male = 30.0 ± 9.2%; CI female = 51.0 ± 8.9%, CI male = 44.0 ± 9.1%; CI-R female = 47.5 ± 5.7%, CI-R male = 23.5 ± 2.3% of baseline; two-way repeated measures ANOVA: CTL female vs male *p* = 0.84; PI female vs male *p* = 0.057; CI female vs male *p* = 0.083; CI-R female vs male: interaction: F_(10,33)_ = 0.07, *p* = 0.999, sex: F_(1,33)_ = 223.53, *p* < 0.01, time: F_(10,33)_ = 0.22, *p* = 0.993). These results indicate that, in general, there is no differences in LTP development except for the resocialization group, where males had significantly lower magnitude of LTPs compared to females. Comparisons within groups are shown in the SI section (Fig. S6e,f in SI). These results indicate that LTP develops normally in females at any stress condition compared to CTL. However, males have subtle (PI group) and significant (CI-R group) lower LTP magnitude in comparison to females, indicating that some synaptic plasticity mechanisms are premature affected.

### Effects of long-term chronic social isolation stress on synaptic-related proteins in hypothalamus, hippocampus and prefrontal cortex

**Figure 6:**
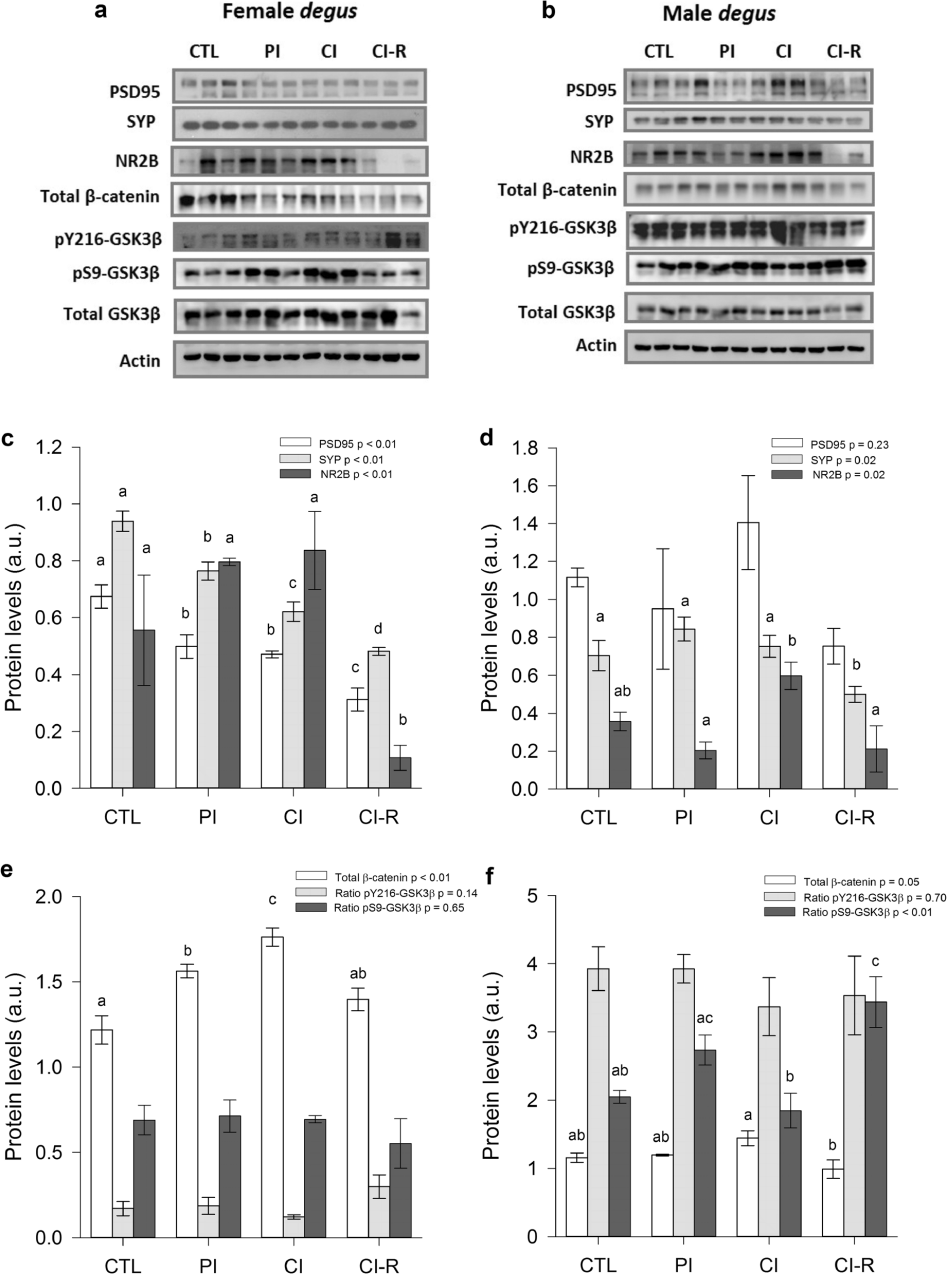
Biochemical analysis of hypothalamic synaptic and canonical Wnt signalling proteins. Western blot analysis for (**a**) female degus (**b**) male degus. Densitometric analysis hypothalamic PSD95, SYP, and NR2B proteins of (**c**) female degus (**d**) male degus. Densitometric analysis of hypothalamic total β-catenin, ratio pY216-GSK3β, and ratio pS9-GSK3β of (**e**) female degus (**f**) male degus. Data were analysed statistically using one-way ANOVA, with the p-value indicated at the top of each figure. Different letters above bars show statistical differences between the same protein across the stress treatments (Fisher’s LSD post hoc test). Results are expressed as mean ± SE (n = 3). a.u: arbitrary units. Control (CTL), Partial Isolation (PI), Chronic Isolation (CI), and Re-socialization (CI-R) treatment groups.

Synaptic proteins dynamics is a crucial factor in the functionality of the synapsis and therefore, in the brain performance. In the pre-synapsis, SYP is one of the most abundant protein of synaptic vesicles and has been described as one modulator of the synaptic vesicle cycle efficiency^71^. In the post- synapsis, PSD95 serves as scaffold for the clustering of several receptors in the active zone of the synapsis. It plays an important role in synaptic plasticity and in the stabilization of synaptic changes during LTP^72^. The functional properties of the postsynaptic receptors usually depend on their subunit composition. NMDA receptors (NMDAR) containing subunit 2B (NR2B) are a crucial component in the plasticity process^73^; its presence can determine the equilibrium toward more or less LTP. Therefore, the effect of SIS conditions on these proteins were studied. In the hypothalamus of female degus, PSD95 levels differed across stress treatment [F_(3,8)_ = 17.19, *p* < 0.01]. In particular, all stress conditions were significantly lower compared to the CTL group, with the CI-R group having the lowest level (Fig. 6c); whereas in males, the PSD95 did not significantly differ among stress treatments (*p* = 0.23; Fig. 6d). The expression of SYP protein was significantly affected by stress treatment in both female and male degus [F_(3,8)_ = 42.58, *p* < 0.01 and F_(3,8)_ = 5.49, *p* = 0.02, respectively]. Similar, to PSD95 in females, SYP expression decreased under stress conditions, with the CI-R group having the lowest value in both sexes (Fig. 6c,d). In the case of NR2B, the expression in the hypothalamus of female and male degus was significantly affected by stress treatment [F_(3,8)_ = 7.65, *p* < 0.01 and F_(3,8)_ = 5.54, *p* = 0.02, respectively]. Particularly, CI-R females had significantly lower levels of NR2B compared to the other groups (Fig. 6c); whereas in males, NR2B increased significantly in CI and decreased in CI-R group to basal levels (Fig. 6d).

**Figure 7:**
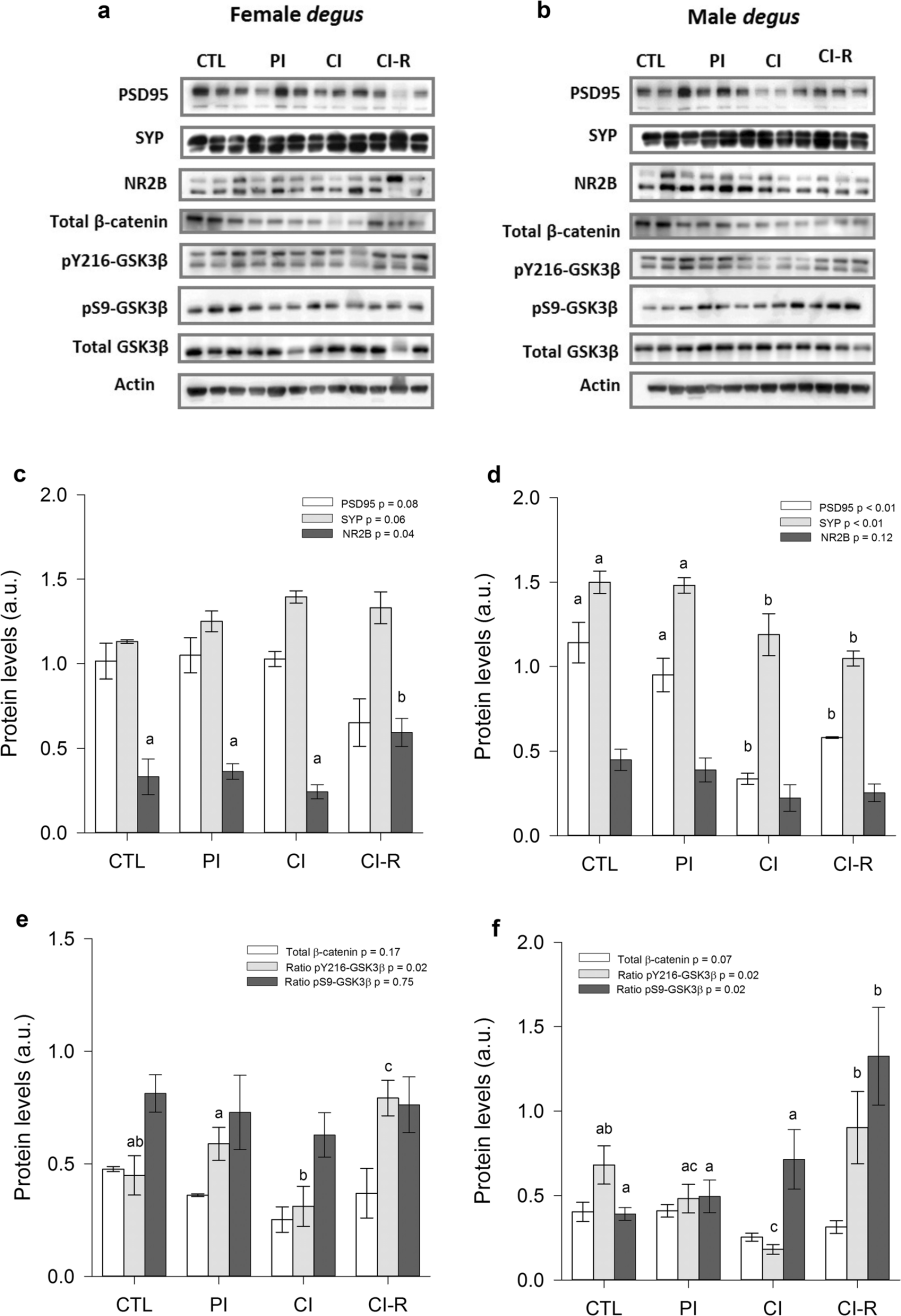
Biochemical analysis of hippocampal synaptic and canonical Wnt signalling proteins. Western blot analysis for (**a**) female degus (**b**) male degus. Densitometric analysis of hippocampal PSD95, SYP, and NR2B proteins of (**c**) female degus (**d**) male degus. Densitometric analysis of hippocampal total β-catenin, ratio pY216-GSK3β, and ratio pS9-GSK3β of (**e**) female degus (**f**) male degus. Data were analysed statistically using one-way ANOVAs, with the p-value indicated at the top of each figure. Different letters above bars show statistical differences between the same protein across stress treatments (Fisher’s LSD post hoc test). Results are expressed as mean ± SE (n = 3). a.u: arbitrary units. Control (CTL), Partial Isolation (PI), Chronic Isolation (CI), and Re-socialization (CI-R) treatment groups.

On the other hand, hippocampal lysates of female degus revealed no significant differences in postsynaptic protein PSD95 with stress treatment (*p* = 0.08; Fig. 7c]. Instead, in males we observed a significant effect of the stress treatment [F_(3,8)_ = 20.76, *p* < 0.01] with CI and CI-R groups having significantly lower values compared with the CTL and PI groups (Fig. 7d). Like PSD95, female degu SYP protein expression (Fig. 7c) did not significantly differ with stress treatment (*p* = 0.06). Whereas in males we observed a significant effect of the stress treatment [F_(3,8)_ = 8.26, *p* < 0.01], with significantly reduced in SYP levels CI and CI-R compared to CTL and PI groups (Fig. 7d). Hippocampal expression of NR2B in females significantly differed with stress treatment [F_(3,8)_ = 4.09; *p* = 0.04], where CI-R group had higher values of this protein compared to other groups (Fig. 7c); whereas no differences within male groups were detected (*p* = 0.12; Fig. 7d).

**Figure 8:**
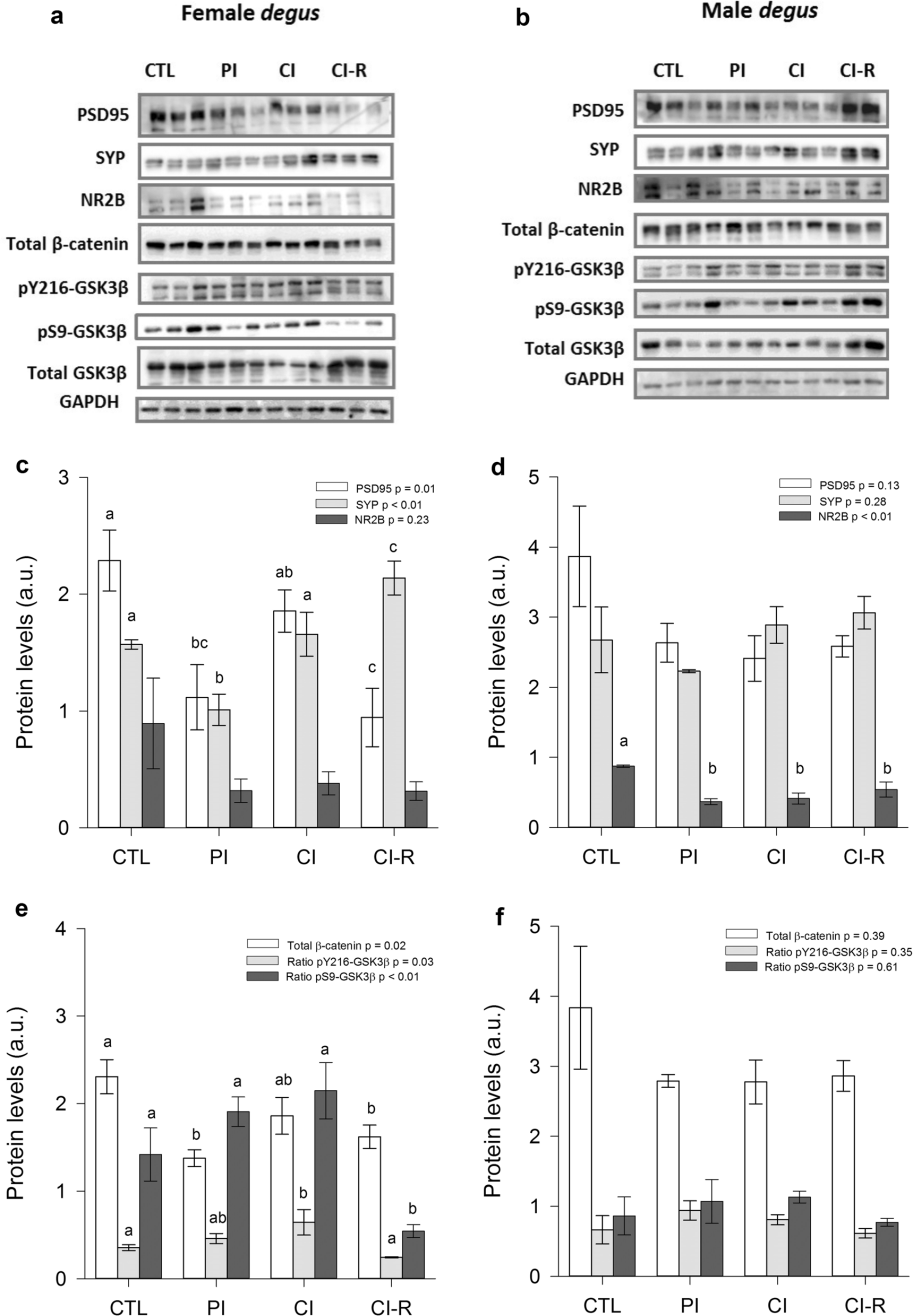
Biochemical analysis of prefrontal cortex (PFC) synaptic and canonical Wnt signalling proteins. Western blot analysis for (**a**) female degus (**b**) male degus. Densitometric analysis of PSD95, SYP, and NR2B proteins of (**c**) female degus PFCs (**d**) male degus PFCs. Densitometric analysis of total β-catenin, ratio pY216-GSK3β, and ratio pS9-GSK3β of (**e**) female degus PFCs (**f**) male degus PFCs. Data were analysed statistically using one-way ANOVAs, with p-values indicated at the top of each figure. Different letters above bars show statistical differences between the same protein across stress treatments (Fisher’s LSD post hoc test). Results are expressed as mean ± SE (n = 3). a.u: arbitrary units. Control (CTL), Partial Isolation (PI), Chronic Isolation (CI), and Re-socialization (CI-R) treatment groups.

In PFC of female degus, a one-way ANOVA for PSD95 protein levels revealed a significant effect of stress treatment [F_(3,8)_ = 6.59, *p* = 0.01], where PI and CI-R females had lower levels of PSD95 compared to the CTL and CI group (Fig. 8c). Conversely, in males PSD95 expression levels did not significantly changed with stress treatment (*p* = 0.13; Fig. 8d). For SYP protein levels in female degus, we found a statistically significant effect of stress treatment [F_(3,8)_ = 11.22, *p* < 0.01], where PI had the lower value and CI-R females had higher levels of this protein compared to CTL and CI females (Fig. 8c). Conversely, male showed not significantly differences across stress treatments (*p* = 0.28; Fig. 8d). For the NR2B expression in PFC, female did not significantly vary with stress treatments (*p* = 0.23; Fig. 8c); however, male degus showed a significant effect of stress treatment [F_(3,8)_ = 10.48, *p* < 0.01], with males under different degrees of social isolation stress showing lower values of this protein when compared to CTL males (Fig. 8d). Taken together, these results indicate that synaptic proteins were affected differently by the stress treatment, sex and brain region (See Fig. S8 in SI for a schematic summary of the dynamic of these proteins).

### Effects of long-term chronic social isolation stress on canonical Wnt signalling proteins in hypothalamus, hippocampus and prefrontal cortex

Canonical Wnt signalling pathway plays a major role in adult brain morphogenesis, differentiation and synaptic plasticity^74^. Its activation involve the inhibition of GSK3β and the consequent accumulation of β-catenin, which is required for the transcription of context-dependent genes associated with cell growth, differentiation and cell communication^75^. Therefore, we determined whether SIS can alter some crucial components of canonical Wnt signalling proteins, like as total β-catenin, total GSK3β, pY216-GSK3β (activated), and pS9-GSK3β (inhibited) in hypothalamus, hippocampus, and PFC of SIS degus.

For the total β-catenin levels in the hypothalamus of female degus, we found a statistically significant effect of stress treatment [F_(3,8)_ = 21.88, *p* < 0.01], where β-catenin was higher in PI and CI compared to CTL (Fig. 6e). Whereas in males, almost significant differences with stress treatment [F_(3,8)_ = 4.02, *p* = 0.05], with lower levels of this protein in the CI males compared with the CI-R group (Fig. 6f). In terms of hypothalamic pY216-GSK3β ratio, there was no significant effect of the stress treatment in both female and male degus (*p* = 0.14 and *p* = 0.70, respectively; Fig. 6e,f). In the case of pS9-GSK3β ratio, no changes along stress treatments were observed in females (*p* = 0.65; Fig. 6e); while in males, we found a statistically significant effect of stress treatment [F_(3,8)_ = 8.07, *p* < 0.01]. Further comparisons showed that CI group had significantly lower levels, and CI-R males showed higher levels compared to CTL and CI males (Fig. 6f).

Meanwhile in the hippocampus, we found no changes in total β-catenin levels across stress treatments, both in female and male degus (*p* = 0.17 and *p* = 0.07, respectively; Fig. 7e,f). In terms of hippocampal pY216-GSK3β ratio, there was a significant effect of stress treatment in female and male degus [F_(3,8)_ = 6.20, *p* = 0.02 and F_(3,8)_ = 5.63, *p* = 0.02, respectively]. Additional analysis showed that CI females had lower levels and CI-R females had higher levels of this protein compared to other stress treatments (Fig. 7e). A similar pattern was also observed in males, with a dramatic decrease in CI group compared to CTL and CI-R groups (Fig. 7f). In the case of pS9-GSK3β ratio, we observed no changes across stress treatments in females (*p* = 0.75; Fig. 7e), while in males there was a significant effect of stress treatment [F_(3,8)_ = 5.57, *p* = 0.02], with CI-R group showing higher values compared to the other stress treatment groups (Fig. 7f).

In the PFC of female degus, protein levels of total β-catenin levels showed a significant effect of stress treatment [F_(3,8)_ = 5.74, *p* = 0.02]. Further analysis showed that PI and CI-R female degus had lower levels of this protein compared with CTL group (Fig. 8e); whereas no differences between stress treatments were found in male degus (*p* = 0.39; Fig. 8f). In terms of pY216-GSK3β ratio for female degus, a significant effect of the stress treatment was found [F_(3,8)_ = 4.48, *p* = 0.03], where CI females had significantly higher values compared with CTL and CI-R (Fig. 8e), whereas male degus did not show differences among stress treatment (*p* = 0.35; Fig. 8f). On the other hand, the pS9-GSK3β ratio showed a significant effect of stress treatment in female degus [F_(3,8)_ = 0.75, *p* < 0.01], with lower levels of this protein in the CI-R females compared to the other groups (Fig. 8e). Instead, there were no significant differences between treatments in male degus (*p* = 0.61; Fig. 8f). Taken together, these results suggest that the SIS induce several effects on the canonical Wnt signalling pathway which were treatment-, sex- and brain region-dependent (See Fig. S8 in SI for a schematic summary of the dynamic of these proteins).

## Discussion

The purpose of this study was to investigate the relationship between behavioural-cognitive alterations, physiological effects and brain molecular changes induced by long-term chronic SIS. The study involves female and male degus under different stress treatments, from post-natal and post-weaning until adulthood. More importantly, in this paper we also evaluated if social buffering (re-socialization) would be able to mitigate reactive stress responses in long-term chronic SIS degus. Degus are long-lived rodents that under captivity conditions lives in average 7 years. Then, despite that CI-R group stayed additional 6-months for the re-socialization purpose, we considered that all our treatments are comparable in time. According to previous studies in our laboratory, we know that degus can start experimenting biochemical changes and impairment in cognitive performance associated with normal ageing, only > 3 years old^47,58,76–78^. Our treatments were performed during the first 2 years of life, when all animals are considered mature, healthy and biologically similar.

### Long-term chronic social isolation stress effects on Hypothalamic‐pituitary‐adrenal (HPA) axis

We evaluated the endocrine stress response through measurement of baseline and stress-induced cortisol levels, and the assessment of negative feedback efficacy. While the negative feedback data are in accordance with previous studies, the basal and stress-induced cortisol findings are not what we expected. Although baseline GC levels can be used as indicators of animal's health and condition^79^, in many studies basal GC levels were not different in socially isolated animals compared to social-housed counterparts^50,80–83^. The fact that we did not detect an increase in basal and stress-induced plasma cortisol in CI degus suggest the adaptation to the isolation experience. Indeed, negative feedback is an integral characteristic of the HPA-axis, and a hyper-reactive stress response is often typified by weak negative feedback^84,85^. Similar to our study, other reports found that rats exposed to early-life social isolation had unchanged baseline and stress-induced CORT, but a reduced negative feedback strength. Furthermore, rats regained their typical GC negative feedback regulation after re-socialization^83^. Only one study has examined the effect of long-lasting SIS (6½ months) in female degus^46^. However, while the authors showed SIS affected body weight gain (a reliable indicator of chronic stress), there was no effect on basal cortisol levels. Unlike previous studies that showed that social isolation appears to affect HPA axis activity in a sex-specific manner^81,82,86^, our study fails to show any differences between female and male degus in GC levels and negative feedback efficacy.

### Long-term chronic social isolation stress effects on cognitive performance

Impairment of the negative feedback loop for the HPA axis has been related with cognitive deficits^87^. We first performed an exploratory multivariate analysis. For spatial memory, our results showed that both female and male degus under CI treatment formed a separate group from the CTL groups. Interestingly, the behaviour of CI-R female and male degus was similar to CTL degus. For learning performance, we found that SIS only significantly impacted male degus, suggesting that the effect of long-term chronic SIS is more pronounced in male than female degus.

Similar results have been reported in other rodent studies^88–90^. By using univariate analysis, we observed that working episodic memory (measured by RI in the NLR/NOR task) was impaired in both female and male degus under CI treatment, and more importantly, re-socialization was able to restore both the working memory and the innate rodent predilection for novel experiences (as exploration of new objects) in both sexes. In this regard, previous works using mice and rats have described deficits of spatial memory resulting from social isolation^5,91–93^. Whether re-socialization can restore these spatial memory deficits, however, is still controversial. For example, Ouchi et al.^94^ found that socially isolated mice showed spatial attention deficits, and that re-socialization failed to attenuate these spatial attention deficits. In contrast, in other studies, re‐socialized mice with stress‐associated mental disorders were able to completely recover spatial/non‐spatial cognitive deficits caused by social isolation^95^.

To examine the impact of long-term SIS on the ability to learn and remember under stressful conditions, we further examined the ability of female and male degus to find the escape hole in the Barnes maze test. Our results suggest sex differences in spatial learning during acquisition (i.e., training period) of the Barnes maze. In general, male degus took less time to find the escape hole during the training period compared to females. Moreover, CI male degus took significantly longer time to find the escape hole compared to other groups, whereas no changes were observed in female degus. Similarly, male degus made less reference memory errors than females. During the training period, both CI female and male degus made more reference memory errors than other groups. Whereas, the number of working memory errors were similar between both sexes, and again, both CI females and males registered more working memory errors than the other groups. We also observed sex-dependent differences in navigation strategies used to find the escape hole. In general, CTL female degus preferred a serial-search strategy compared to CTL males who used more frequently a spatial-strategy. Both PI female and male degus preferred a serial- and random-strategies, whereas CI females and males more commonly adopted a random-strategy. Interestingly, CI-R females and males changed to a serial and spatial-search strategies. Taken together, our data demonstrated that the effect of SIS on cognition during acquisition period of the Barnes maze, is sex-dependent. More importantly, during training phase CI-R degus behaved similarly to CTL, confirming that 6-months of re-socialization can restore normal behaviour.

During the testing phase, we observed that females took more time to find the escape hole than males, however, female degus showed no changes across treatments; whereas CI male degus required about twice the amount of time to locate the escape hole compared with the other groups. Furthermore, CI and CI-R female degus made more reference and working memory errors compared to CTL females, whereas no changes were observed in males. For the strategy taken by degus to find the escape hole we again observed a sex-dependent strategy. In general, female degus across the stress treatments preferred a serial-search strategy to navigate across the Barnes maze. Instead, CI males applied mostly a random strategy, opposite to CTL males who typically applied spatial- and serial-search strategies. Interestingly, CI-R males alternated between serial- and spatial-search strategies, more similar to CTL males.

Most of the studies on spatial navigation tasks using water and/or radial maze point out that male mice and rats made fewer reference and working memory errors than females^96,97^, whereas other demonstrated female superiority in working memory^98^, and others failed to show sex differences in rats^99^. Moreover, there are few studies that have investigated sex differences in the Barnes maze test^100–103^. Only one of them compared sex differences in spatial learning and memory in degus^61^, showing that female degus use a spatial strategy, whereas males preferably apply either serial, random or opposite strategies in the Barnes maze test. Our study revealed that, in general, the most common strategy used by female degus across treatments was the serial-search strategy; whereas, in males we observed a spatial- and serial-search strategies in CTL, a random-search patterns in CI, and serial- and spatial-search strategies in PI and CI-R treatments. Popovic et al.^61^ suggested that gender differences in the type of strategy are due to male preferences for navigation and exploring different ways to find the escape hole, while females always use the fastest exit strategy after they first learn how to escape. Likewise, these authors also indicated that during acquisition period (i.e., training phase) of the Barnes maze female degus showed superiority in both reference and working memory, opposite to our findings. Moreover, these authors found no significant differences between female and male degus during the retrieval phase (i.e., test phase), suggesting that there were gender differences in long-term memory^61^. A plausible explanation for the differences between our data and previous degus studies can be attributed to the possibility that long-term social isolation exacerbated the impairment of spatial memory during the consolidation phase (i.e., test phase). In support to our findings, the study of Pereda-Perez et al.^50^ in adult female degus showed that long-term social isolation (6½ months) impaired the contextual fear memory (a hippocampal- dependent task), reduced hippocampal synaptic levels, and induced a specific shrinkage of CA1.

Regarding to this, our electrophysiological analysis showed that PI and CI-R males have problems in sustain synaptic plasticity in slices, a kind of memory that involves spatial learning. This indicates that the retrieval of memory mechanisms in this circuit was affected in males only when they were early exposed to isolation (PI); same deficits were again evidenced when animals were chronically isolated and then re-socialized (CI-R). This suggests that in both cases the mechanisms affected are the same, there were probably early-caused, and that emotional contact with pairs is the trigger that retrieves the manifested differences.

### Long-term chronic social isolation stress effects and its consequences for brain function

Long-term storage of memories is known to be dependent on de novo protein synthesis, possibly involving changes in existing brain circuitry and affecting synaptic plasticity^104^. Exposure to an enriched environment is beneficial to the structure and function of the brain^105^. Social buffering can act as an environmental enrichment, improving the storage of new information and experience-dependent behavioural modifications^104^.

Chronic stress increases HPA-axis excitability, contributing to a chronic stress-induced hyper-reactivity state in the brain^106^. Glutamatergic neurotransmission is an important component in the regulation of neuroendocrine function in the hypothalamus, where NMDAR has a role inducing hormone release^107^. Further studies indicate that chronic stress increases the activity of hippocampal NMDAR, thus causing learning and memory impairment^108^. In our study, we observed different changes in the level of synaptic protein along the experimental treatments, both in females and males. In the hypothalamus of female degus, a presynaptic protein like SYP and a postsynaptic protein like PSD95 reduced their levels across stress treatments. Together with the reduction of NR2B levels in the CI-R group it suggests that hypothalamic synaptic structure is permanently modified by SIS. A similar effect was obtained in males, where SYP and NR2B had lower levels in CI-R than CI degus^104^. In general, these results indicate that social state affect the hypothalamus similarly in female and male degus, specifically because resocialization cannot restore these protein levels affected by SIS. Normal hypothalamic development differs between females and males, resulting in a sexually dimorphic hypothalamic structure with specific functions. Therefore, it is expected that environmental challenges such as SIS during this critical period may differentially affect sexes^109^. As we observed, despite few differences, SIS appears to have a great impact at the level of synaptic protein components in both sexes.

GC receptors are present in the hippocampus at relatively high concentrations, thus playing an important role in memory and complex behaviours^110^. In the hippocampus of female degus, both PSD95 and SYP levels did not change across treatments, however NR2B was upregulated in CI-R group. Hence, synaptic proteins of females appear to be more conserved despite differential stress exposure. This is interesting, because it can be related with our physiological measurements, where LTP measured in hippocampal slices of females was normally developed and not affected by stress treatments. However, males were different; LTP was significantly lower in male CI-R group compared to females CI-R, showing a sustained decrease and indicating that long-term potentiation fails to consolidate in time. Interestingly, PI males are barely out of significance from females PI, demonstrating that early life stress can also affect memory-related processes. At the level of proteins, we found low levels of PSD95 in male CI-R, which agree with the lack of LTP stability, and low levels of SYP in CI and CI-R males. Therefore, males appear to be more susceptible to stressful conditions. Nevertheless, males display other physiological compensations to achieve synaptic activity properly. Using electrophysiology, we found that I-O slope curves are higher in PI and CI-R males compared to females. Correlating the number of activated axons by evoked response, we found that males are by far more efficient in synaptic transmission than females, in all conditions but mainly in CI and CI-R groups. This means that less recruited axons are enough to evoke bigger response. This compensatory effect could have molecular basis to still be determined, such as a greater number of postsynaptic receptors (i.e. GluRs, NR2A) and changes in presynaptic terminals (i.e. number of vesicles released, larger active sites, etc.)^111–113^. In any case, in males these effects are insufficient to support LTP normally as in females, and probably includes defects in LTP molecular machinery that re-socialization cannot compensate. Long-term social isolation in adult female degus has been shown to reduce some synaptic proteins and the size of CA1 in hippocampus^50^. Other data suggests that chronic stress and corticosterone exposure lead to loss of spines, alterations in LTP/LTD, and impairment in memory tasks^110^. Therefore, more studies should be made to determine the wide range of molecular and structural effects in this brain region.

Chronic stress or chronic administration of GCs produces dendritic remodelling in pyramidal neurons of the PFC, and causes NMDAR activation which are highly represented in this zone^104,114^. We analysed the PFC region and found that in females both PSD95 and SYP levels were decreased in PI, increased in CI, but only SYP and not PSD95 remained elevated in the CI-R group, suggesting that pre- and post-synaptic terminals at PFC respond differently to SIS. While NR2B did not significantly change across treatments in females, it was the only protein that significantly changed in males, where decreases in all treatment groups compared to CTL. These alterations in the PFC could be involved in different navigation tasks strategies previously observed in CI and CI-R groups.

In the nervous system, the canonical Wnt signalling pathway has been predominantly linked to embryonic development, but within the past few decades it has become clear that it also plays a major role in adult brain morphogenesis, differentiation, and synaptic activity^75,115–118^. In the hypothalamus, this pathway has been related to energy balance regulation and neurogenesis^115,117^. In our study, the hypothalamus analyses displayed that only β-catenin transiently increases in PI and CI, returning to CTL levels in CI-R female degus. In males, the reduction in β-catenin levels coincides with the increase of inactive GSK3β levels at CI-R group. These results suggest that, independent of the sex, canonical Wnt/β-catenin-related target genes are being expressed under SIS and are returned to control levels under re-socialization.

In the hippocampus, no changes in β-catenin but a significant modulation of the phosphorylation levels of GSK3β were found. The active form of this protein decreases at CI and increases at CI-R degus in both sexes. Instead, the inactive GSK3β form increased only in CI-R males, as it was also found in hypothalamus. These data suggest that canonical Wnt signalling is activated during CI to upregulate the downstream signalling, maintaining the hippocampal cellular homeostasis. During re-socialization, the active and inhibited GSK3β forms stayed high in males, suggesting that canonical Wnt signalling activation reach an equilibrium.

In the PFC of females, β-catenin follows the same pattern than PSD95, a correlation that has been previously observed^119^; and also correlates with the presynaptic SYP protein during early stress (PI). This could be relevant for PFC, where most working memory processes has been allocated^120^, suggesting that β-catenin is modulating synaptic proteins in PFC. While in males, no significant changes at any of these proteins were detected, on females the state of active GSK3β transits from being active during CI, returning to basal levels during re-socialization. Instead, during re-socialization both β-catenin and inhibited GSK3β reached their lower levels, indicating that canonical Wnt signalling can be activated again with re-socialization. Altogether these data indicate that Wnt signalling proteins are in a tightly regulated equilibrium where activation increases across SIS in a brain structure- and sex-dependent manner.

Recent in vitro studies have demonstrated that canonical Wnt signalling is able to modulate the levels of several plasticity-related proteins as Ca^+2^/Calmodulin-dependent kinase II (CaMKII) and cAMP response element-binding protein (CREB). Specifically, canonical Wnt signalling plays a key role in controlling neuron activity-regulated Brain-Derived Neurotrophic-Factor (BDNF) expression^121^. Additionally, canonical Wnt signalling, through GSK-3β, mediate BDNF’s effects as a master regulator of synaptic plasticity, neuronal differentiation and neuroprotection^122^. Further experiments analysing BDNF and CREB levels should be performed to reveal plasticity processes underling our results.

As previously discussed, it is possible that discrepancies between previous studies might be due to differences in social isolation protocols; shorter isolation periods (3-months) may not be enough to induce changes in synaptic transmission compared to controls^105^. Instead, in our study long-term social isolation and/or buffering protocols may induce major changes on synaptic transmission, synaptic proteins, and consequently on the cellular mechanisms associated with it. Altogether, we found that although no sex-dependent differences were observed in endocrine analysis, SIS impaired the negative feedback loop for the HPA axis. This effect can be related to the deficit in learning and memory processes observed in chronically stressed female and male degus. Moreover, several differences at the level of synaptic activity could be directly related with many of the behavioural differences observed across females and males. In accordance, optimization of synaptic transmission does not necessarily correlate into improved synaptic plasticity, and females appear to be more stable than males in terms of plasticity mechanisms, which remain undisturbed by stress. Instead, males appear optimizing their synaptic transmission in order to accommodate to stress-caused disturbances. Regarding protein measurements, is interesting to realise that pre- and post-synaptic proteins appear to behave in similar features within females or males, and mainly affected negatively by stress treatments. Another interesting correlation appears between CI and CI-R, where whenever the first increases protein level, the last do the opposite, most of the time recovering up to control levels (Fig. S8 in SI). Therefore, synaptic and canonical Wnt signalling proteins from three main areas related to cognition, learning and socialization (PFC, hippocampus, and hypothalamus, respectively), displayed both transient and permanent changes during social isolation and re-socialization.

## Supplementary information

Supplementary information 1.

Supplementary information 2.

## References

[1] Beery AK, Kaufer D (2015). Stress, social behavior, and resilience: insights from rodents. Neurobiol. Stress.

[2] Neumann ID (2009). The advantage of social living: brain neuropeptides mediate the beneficial consequences of sex and motherhood. Front. Neuroendocrinol..

[3] Buwalda B (2013). Adolescent social stress does not necessarily lead to a compromised adaptive capacity during adulthood: a study on the consequences of social stress in rats. Neuroscience.

[4] Mumtaz F (2018). Neurobiology and consequences of social isolation stress in animal model-A comprehensive review. Biomed. Pharmacother..

[5] Bianchi M (2006). Isolation rearing induces recognition memory deficits accompanied by cytoskeletal alterations in rat hippocampus. Eur. J. Neurosci..

[6] Fone KC, Porkess MV (2008). Behavioural and neurochemical effects of post-weaning social isolation in rodents-relevance to developmental neuropsychiatric disorders. Neurosci. Biobehav. Rev..

[7] Zhao X (2009). Isolation rearing induces social and emotional function abnormalities and alters glutamate and neurodevelopment-related gene expression in rats. Prog. Neuropsychopharmacol. Biol. Psychiatry.

[8] Lapiz MD (2003). Influence of postweaning social isolation in the rat on brain development, conditioned behavior, and neurotransmission. Neurosci. Behav. Physiol..

[9] Westenbroek C (2004). Chronic stress and social housing differentially affect neurogenesis in male and female rats. Brain Res. Bull..

[10] Schoenfeld TJ, Gould E (2011). Stress, stress hormones, and adult neurogenesis. Exp. Neurol..

[11] Liu C (2016). Altered structural connectome in adolescent socially isolated mice. Neuroimage.

[12] Smith SM, Vale WW (2006). The role of the hypothalamic-pituitary-adrenal axis in neuroendocrine responses to stress. Dialogues Clin. Neurosci..

[13] Bale TL, Epperson CN (2015). Sex differences and stress across the lifespan. Nat. Neurosci..

[14] Sandi C, Haller J (2015). Stress and the social brain: behavioural effects and neurobiological mechanisms. Nat. Rev. Neurosci..

[15] Wommack JC (2004). Behavioural and neuroendocrine adaptations to repeated stress during puberty in male golden hamsters. J. Neuroendocrinol..

[16] Herman JP (2016). Regulation of the hypothalamic-pituitary-adrenocortical stress response. Compr. Physiol..

[17] Lupien SJ, McEwen BS (1997). The acute effects of corticosteroids on cognition: integration of animal and human model studies. Brain Res. Brain Res. Rev..

[18] Lupien SJ (2009). Effects of stress throughout the lifespan on the brain, behaviour and cognition. Nat. Rev. Neurosci..

[19] de Kloet ER, Oitzl MS, Joels M (1999). Stress and cognition: are corticosteroids good or bad guys?. Trends Neurosci..

[20] McEwen BS (1998). Protective and damaging effects of stress mediators. N. Engl. J. Med..

[21] McEwen BS (2001). Plasticity of the hippocampus: adaptation to chronic stress and allostatic load. Ann. N. Y. Acad. Sci..

[22] Avital A, Richter-Levin G (2005). Exposure to juvenile stress exacerbates the behavioural consequences of exposure to stress in the adult rat. Int. J. Neuropsychopharmacol..

[23] De Kloet ER (1998). Brain corticosteroid receptor balance in health and disease. Endocr Rev.

[24] Myers B, McKlveen JM, Herman JP (2012). Neural regulation of the stress response: the many faces of feedback. Cell Mol. Neurobiol..

[25] Kovacs KJ, Foldes A, Sawchenko PE (2000). Glucocorticoid negative feedback selectively targets vasopressin transcription in parvocellular neurosecretory neurons. J. Neurosci..

[26] Herman JP (2012). Neural regulation of the stress response: glucocorticoid feedback mechanisms. Braz. J. Med. Biol. Res..

[27] Hawkley LC (2012). Effects of social isolation on glucocorticoid regulation in social mammals. Horm. Behav..

[28] Arnett MG (2015). Genetic Approaches to Hypothalamic-Pituitary-Adrenal Axis Regulation. Neuropsychopharmacology.

[29] Liu D (2000). Influence of neonatal rearing conditions on stress-induced adrenocorticotropin responses and norepinepherine release in the hypothalamic paraventricular nucleus. J. Neuroendocrinol..

[30] Ladd CO (2004). Long-term adaptations in glucocorticoid receptor and mineralocorticoid receptor mRNA and negative feedback on the hypothalamo-pituitary-adrenal axis following neonatal maternal separation. Biol. Psychiatry.

[31] Liu PZ, Nusslock R (2018). How Stress Gets Under the Skin: Early Life Adversity and Glucocorticoid Receptor Epigenetic Regulation. Curr.. Genomics.

[32] Liu D (1997). Maternal care, hippocampal glucocorticoid receptors, and hypothalamic-pituitary-adrenal responses to stress. Science.

[33] Novais A (2017). How age, sex and genotype shape the stress response. Neurobiol. Stress.

[34] Babygirija R (2012). Social interaction attenuates stress responses following chronic stress in maternally separated rats. Brain Res..

[35] Nishi M, Horii-Hayashi N, Sasagawa T (2014). Effects of early life adverse experiences on the brain: implications from maternal separation models in rodents. Front. Neurosci..

[36] Gilles YD, Polston EK (2017). Effects of social deprivation on social and depressive-like behaviors and the numbers of oxytocin expressing neurons in rats. Behav. Brain Res..

[37] de Kloet ER, Schmidt M, Meijer OC (2005). Chapter 3.1 Corticosteroid receptors and HPA-axis regulation. Tech. Behav. Neural Sci..

[38] Weaver IC (2004). Epigenetic programming by maternal behavior. Nat. Neurosci..

[39] Hennessy MB, Maken DS, Graves FC (2000). Consequences of the presence of the mother or unfamiliar adult female on cortisol, ACTH, testosterone and behavioral responses of periadolescent guinea pigs during exposure to novelty. Psychoneuroendocrinology.

[40] Panksepp J, Panksepp JB (2013). Toward a cross-species understanding of empathy. Trends Neurosci..

[41] Berardi A (2014). An updated animal model capturing both the cognitive and emotional features of post-traumatic stress disorder (PTSD). Front. Behav. Neurosci..

[42] Rincon-Cortes M, Sullivan RM (2014). Early life trauma and attachment: immediate and enduring effects on neurobehavioral and stress axis development. Front. Endocrinol. (Lausanne).

[43] de Waal FBM, Preston SD (2017). Mammalian empathy: behavioural manifestations and neural basis. Nat. Rev. Neurosci..

[44] Levy DR, Yizhar O (2018). Stress and sociability. Nat. Neurosci..

[45] Blanchard RJ, McKittrick CR, Blanchard DC (2001). Animal models of social stress: effects on behavior and brain neurochemical systems. Physiol. Behav..

[46] Colonnello V (2011). *Octodon degus*. A useful animal model for social-affective neuroscience research: basic description of separation distress, social attachments and play. Neurosci. Biobehav. Rev..

[47] Rivera DS, Inestrosa NC, Bozinovic F (2016). On cognitive ecology and the environmental factors that promote Alzheimer disease: lessons from Octodon degus (Rodentia: Octodontidae). Biol. Res..

[48] Lee TM (2004). Octodon degus: a diurnal, social, and long-lived rodent. ILAR J..

[49] Mahoney MM (2011). Characterization of the estrous cycle in Octodon degus. Biol. Reprod..

[50] Pereda-Perez I (2013). Long-term social isolation in the adulthood results in CA1 shrinkage and cognitive impairment. Neurobiol. Learn. Mem..

[51] Braun K (2003). Influence of parental deprivation on the behavioral development in Octodon degus: modulation by maternal vocalizations. Dev. Psychobiol..

[52] Braun K (2011). The prefrontal-limbic system: development, neuroanatomy, function, and implications for socioemotional development. Clin. Perinatol..

[53] Kenagy GJ, Place NJ, Veloso C (1999). Relation of glucocorticosteroids and testosterone to the annual cycle of free-living degus in semiarid central Chile. Gen. Comp. Endocrinol..

[54] Bauer CM (2016). Postnatal development of the Degu (*Octodon degus*) endocrine stress response is affected by maternal care. J. Exp. Zool. A Ecol. Genet. Physiol..

[55] Romero LM, Reed JM (2005). Collecting baseline corticosterone samples in the field: is under 3 min good enough?. Comp. Biochem. Physiol. A Mol. Integr. Physiol..

[56] Bauer CM (2013). Habitat type influences endocrine stress response in the degu (*Octodon degus*). Gen. Comp. Endocrinol..

[57] Romero LM, Wikelski M (2010). Stress physiology as a predictor of survival in Galapagos marine iguanas. Proc. Biol. Sci..

[58] Rivera DS (2018). Long-term, fructose-induced metabolic syndrome-like condition is associated with higher metabolism, reduced synaptic plasticity and cognitive impairment in *Octodon degus*. Mol. Neurobiol..

[59] Popovic N (2009). Aging and time-of-day effects on anxiety in female *Octodon degus*. Behav. Brain Res..

[60] Barnes CA (1979). Memory deficits associated with senescence: a neurophysiological and behavioral study in the rat. J. Comp. Physiol. Psychol..

[61] Popovic N (2010). Barnes maze performance of Octodon degus is gender dependent. Behav. Brain Res..

[62] Kumazawa-Manita N (2013). Three-dimensional reconstruction of brain structures of the rodent Octodon degus: a brain atlas constructed by combining histological and magnetic resonance images. Exp. Brain Res..

[63] Tarragon E (2014). Memantine prevents reference and working memory impairment caused by sleep deprivation in both young and aged *Octodon degus*. Neuropharmacology.

[64] Inman-Wood SL (2000). Effects of prenatal cocaine on Morris and Barnes maze tests of spatial learning and memory in the offspring of C57BL/6J mice. Neurotoxicol. Teratol..

[65] Jasarevic E (2011). Disruption of adult expression of sexually selected traits by developmental exposure to bisphenol A. Proc. Natl. Acad. Sci. USA.

[66] Jolliffe IT, Cadima J (2016). Principal component analysis: a review and recent developments. Philos. Trans. A Math. Phys. Eng. Sci..

[67] Romero LM (2006). Seasonal changes in hypothalamic-pituitary-adrenal axis sensitivity in free-living house sparrows (*Passer domesticus*). Gen. Comp. Endocrinol..

[68] Reul JM, van den Bosch FR, de Kloet ER (1987). Relative occupation of type-I and type-II corticosteroid receptors in rat brain following stress and dexamethasone treatment: functional implications. J. Endocrinol..

[69] Winer BJ, Brown DR, Michels KM (1991). Statistical Principles in Experimental Design.

[70] Larson J, Munkacsy E (2014). Theta-burst LTP. Brain Res..

[71] Schmitt U (2009). Detection of behavioral alterations and learning deficits in mice lacking synaptophysin. Neuroscience.

[72] Meyer D, Bonhoeffer T, Scheuss V (2014). Balance and stability of synaptic structures during synaptic plasticity. Neuron.

[73] Groc L (2006). NMDA receptor surface mobility depends on NR2A-2B subunits. Proc. Natl. Acad. Sci. USA.

[74] Inestrosa NC, Varela-Nallar L (2015). Wnt signalling in neuronal differentiation and development. Cell Tissue Res..

[75] Oliva CA, Montecinos-Oliva C, Inestrosa NC (2018). Wnt signaling in the central nervous system: new insights in health and disease. Prog. Mol. Biol. Transl. Sci..

[76] Inestrosa NC (2015). Age progression of neuropathological markers in the brain of the chilean rodent *Octodon degus*, a natural model of Alzheimer's Disease. Brain Pathol..

[77] Rivera DS (2016). Andrographolide recovers cognitive impairment in a natural model of Alzheimer’s disease (*Octodon degus*). Neurobiol. Aging.

[78] Cisternas P (2019). Presymptomatic treatment with andrographolide improves brain metabolic markers and cognitive behavior in a model of early-onset Alzheimer’s disease. Front. Cell. Neurosci.

[79] Bonier F (2009). Do baseline glucocorticoids predict fitness?. Trends Ecol. Evol..

[80] Scaccianoce S (2006). Social isolation selectively reduces hippocampal brain-derived neurotrophic factor without altering plasma corticosterone. Behav Brain Res..

[81] Grippo AJ (2007). Social isolation induces behavioral and neuroendocrine disturbances relevant to depression in female and male prairie voles. Psychoneuroendocrinology.

[82] Arndt SS (2009). Individual housing of mice–impact on behaviour and stress responses. Physiol. Behav..

[83] Lukkes JL (2009). Adult rats exposed to early-life social isolation exhibit increased anxiety and conditioned fear behavior, and altered hormonal stress responses. Horm Behav..

[84] Serra M (2005). Social isolation-induced changes in the hypothalamic-pituitary-adrenal axis in the rat. Stress.

[85] Bauer CM (2015). Maternal stress and plural breeding with communal care affect development of the endocrine stress response in a wild rodent. Horm Behav..

[86] Pournajafi-Nazarloo H (2010). Effects of social isolation on mRNA expression for corticotrophin-releasing hormone receptors in prairie voles. Psychoneuroendocrinology.

[87] Issa AM (1990). Hypothalamic-pituitary-adrenal activity in aged, cognitively impaired and cognitively unimpaired rats. J. Neurosci..

[88] Luine V (2016). Sex differences in chronic stress effects on cognition in rodents. Pharmacol. Biochem. Behav..

[89] Luine VN (2007). Chronic stress and neural function: accounting for sex and age. J. Neuroendocrinol..

[90] Luine V (2002). Sex differences in chronic stress effects on memory in rats. Stress.

[91] Voikar V (2005). Long-term individual housing in C57BL/6J and DBA/2 mice: assessment of behavioral consequences. Genes Brain Behav..

[92] McLean S (2008). Isolation rearing impairs novel object recognition and attentional set shifting performance in female rats. J. Psychopharmacol..

[93] Famitafreshi H, Karimian M (2018). Social state influences memory in novel object recognition test through oxidative stress balance in male rats. Open Pharmacol. J..

[94] Ouchi H (2012). Social isolation induces deficit of latent learning performance in mice: a putative animal model of attention deficit/hyperactivity disorder. Behav. Brain Res..

[95] An D (2017). Effects of social isolation, re-socialization and age on cognitive and aggressive behaviors of Kunming mice and BALB/c mice. Anim. Sci. J..

[96] Gresack JE, Frick KM (2003). Male mice exhibit better spatial working and reference memory than females in a water-escape radial arm maze task. Brain Res..

[97] Jonasson Z (2005). Meta-analysis of sex differences in rodent models of learning and memory: a review of behavioral and biological data. Neurosci. Biobehav. Rev..

[98] Bimonte HA (2000). In two species, females exhibit superior working memory and inferior reference memory on the water radial-arm maze. Physiol. Behav..

[99] Schwegler H (1993). Hippocampal morphology and spatially related behavior in Long-Evans and CFY rats. Hippocampus.

[100] Mueller BR, Bale TL (2007). Early prenatal stress impact on coping strategies and learning performance is sex dependent. Physiol. Behav..

[101] Barrett GL (2009). The chronology of age-related spatial learning impairment in two rat strains, as tested by the Barnes maze. Behav. Neurosci..

[102] O'Leary TP, Savoie V, Brown RE (2010). Learning, memory and search strategies of inbred mouse strains with different visual abilities in the Barnes maze. Behav. Brain Res..

[103] O'Leary TP, Brown RE (2009). Visuo-spatial learning and memory deficits on the Barnes maze in the 16-month-old APPswe/PS1dE9 mouse model of Alzheimer's disease. Behav. Brain Res..

[104] Nithianantharajah J, Levis H, Murphy M (2004). Environmental enrichment results in cortical and subcortical changes in levels of synaptophysin and PSD-95 proteins. Neurobiol. Learn. Mem..

[105] Eckert MJ, Abraham WC (2012). Effects of environmental enrichment exposure on synaptic transmission and plasticity in the hippocampus. Curr. Top. Behav. Neurosci..

[106] Flak JN (2009). Chronic stress-induced neurotransmitter plasticity in the PVN. J. Comp. Neurol..

[107] Nijholt I (2000). Modulation of hypothalamic NMDA receptor function by cyclic AMP-dependent protein kinase and phosphatases. J. Neurochem..

[108] Zhou JJ (2018). Enhanced Hypothalamic NMDA Receptor Activity Contributes to Hyperactivity of HPA Axis in Chronic Stress in Male Rats. Endocrinology.

[109] Garcia-Caceres C (2010). Gender differences in the long-term effects of chronic prenatal stress on the HPA axis and hypothalamic structure in rats. Psychoneuroendocrinology.

[110] Cohen JW (2010). Chronic corticosterone exposure alters postsynaptic protein levels of PSD-95, NR1, and synaptopodin in the mouse brain. Synapse.

[111] Massey PV (2004). Differential roles of NR2A and NR2B-containing NMDA receptors in cortical long-term potentiation and long-term depression. J. Neurosci..

[112] Vitureira N, Goda Y (2013). Cell biology in neuroscience: the interplay between Hebbian and homeostatic synaptic plasticity. J. Cell Biol..

[113] Rich MM, Wenner P (2007). Sensing and expressing homeostatic synaptic plasticity. Trends Neurosci..

[114] Martin KP, Wellman CL (2011). NMDA receptor blockade alters stress-induced dendritic remodeling in medial prefrontal cortex. Cereb Cortex.

[115] Benzler J (2013). Hypothalamic WNT signalling is impaired during obesity and reinstated by leptin treatment in male mice. Endocrinology.

[116] Ciani L, Salinas P (2005). WNTs in the vertebrate nervous system: from patterning to neuronal connectivity. Nat. Rev. Neurosci..

[117] Newman EA (2018). Canonical Wnt signaling regulates patterning, differentiation and nucleogenesis in mouse hypothalamus and prethalamus. Dev. Biol..

[118] Oliva CA, Vargas JY, Inestrosa NC (2013). Wnts in adult brain: from synaptic plasticity to cognitive deficiencies. Front. Cell Neurosci..

[119] Ramos-Fernandez E (2019). Wnt-7a stimulates dendritic spine morphogenesis and PSD-95 expression through canonical signaling. Mol. Neurobiol..

[120] Lara AH, Wallis JD (2015). The role of prefrontal cortex in working memory: a mini review. Front. Syst. Neurosci..

[121] Yi H (2011). Expression of brain-derived neurotrophic factor is regulated by the Wnt signaling pathway. NeuroReport.

[122] Yang JW (2015). BDNF promotes the growth of human neurons through crosstalk with the Wnt/beta-catenin signaling pathway via GSK-3beta. Neuropeptides.

