# Supplementary material for: Effects of long-lasting social isolation and re-socialization on cognitive performance and brain activity: a longitudinal study in Octodon degus
Source: Sci Rep. 2020 Oct 27;10:18315. doi: 10.1038/s41598-020-75026-4 (PMC7591540; doi:10.1038/s41598-020-75026-4)
Supplement: Supplementary file 1 — Supplementary information 1. [file 41598_2020_75026_MOESM1_ESM.pdf]

Effects of long-lasting social isolation and re-socialization on cognitive performance and brain activity: a longitudinal study in *Octodon degus*

Daniela S. Rivera, Carolina B. Lindsay, Carolina A. Oliva, Juan Francisco Codocedo, Francisco Bozinovic and Nibaldo C. Inestrosa

SUPPLEMENTARY INFORMATION

Interaction time with a novel object and familiar object during NLR/NOR test

For the time spent with the novel object location during NLR, we found no effect of stress treatment ($p = 0.07$), sex ($p = 0.50$) and no interaction was detected between the two factors ($p = 0.09$; Fig. S2a). Interestingly, when we evaluate the time spent with the familiar object locations during NLR session we found an effect of stress treatment [$F_{(3,96)} = 10.35$, $p < 0.01$], but was not altered by the sex ($p = 0.19$), nor was by the interaction between the two factors ($p = 0.18$; Fig. S2a). Followed analysis showed that CI treatment spent more time interacting with the familiar object compared to the other groups. For the time spent with the novel object during NOR session, we found a statistically significant effect of stress treatment [$F_{(3,96)} = 4.78$, $p < 0.01$], no sex effect ($p = 0.85$), and a significant interaction between the two factors [$F_{(3,96)} = 2.75$, $p = 0.04$; Fig. S2b]. In general stress groups (PI, CI, and CI-R) spent significantly less time interacting with the novel object than CTL. When we analysed the time spent with the familiar object during NOR session, we found a statistically significant effect of stress treatment [$F_{(3,96)} = 6.14$, $p < 0.01$], no sex and interaction effect ($p = 0.17$ and $p = 0.78$, respectively; Fig. S2b). Additional analysis indicates that CI degus spent more time interacting with a familiar object than CTL and CI-R treatments.

Latency to find the escape hole, number of reference and memory errors and the navigation strategy during the training phase on the Barnes maze test

Repeated measures analysis during the training phase, showed that the latency to find the escape hole in female degus showed no effect of stress treatment ($p = 0.11$), time ($p = 0.48$), nor was an interaction was found between the two factors ($p = 0.99$; Table 1 and Fig. S3a). On the other hand, in male degus, the latency to find the escape hole showed a significant effect of treatment [$F_{(3,288)} = 12.23$, $p < 0.01$], time [$F_{(6,288)} = 2.52$, $p = 0.02$], but was not altered by the interaction between the two factors ($p = 0.11$). Further analysis, showed differences between treatments across the seven days of training (Table 1 and Fig. S3b). In particular, CI males had higher latency to find the scape hole across training sessions compared with CTL (day 2, 3, 4, 5, 6, 7), PI (day 1, 2, 3, 7) and CI-R groups (days 2, 3, 4, 5, 7).

We also measured the numbers of reference memory and working memory errors to find the escape hole during the training phase. For the reference memory errors, the repeated measures analysis in female degus resulted in a significant effect of stress treatment [$F_{(3,288)} = 5.80$, $p < 0.01$], a non- significant effect of time ($p = 0.196$), and remained no significantly different across the interaction between both factors ($p = 0.43$). In general CI females made significantly more reference memory errors compared with CTL, PI, and CI-R (Fig. S3c). Moreover, in male degus there was a significant effect of stress treatment [$F_{(3,288)} = 3.27$, $p = 0.02$], a non- significant effect of time ($p = 0.39$), and there was significant interaction between both factors [$F_{(18,288)} = 1.98$, $p = 0.01$]. Similar to females, CI male degus made significantly more reference memory errors compared with CTL and CI-R (Fig. S3d).

On the other hand, for the number of working memory error in female degus, we found a significant effect of stress treatment [$F_{(3,288)} = 5.72$, $p < 0.01$], but was not altered by the time ($p = 0.53$), nor was by the interaction between the two factors ($p = 0.97$). Overall CI female degus had more working memory errors compared with CTL, PI, and CI-R (Fig. S3e). Whereas, in male degus the repeated measures analysis showed a significant effect of stress treatment [$F_{(3,288)} = 3.12$, p =

0.03], a non- significant effect of time ($p = 0.55$), and remained no significantly different across the interaction between both factors ($p = 0.07$). In this case, CI males performed significantly more working memory errors than CTL and CI-R degus (Fig. S3f).

Lastly, throughout the training period, the strategy used by CI females during day 1 consisted of mostly random-serial strategy (54% and 33% respectively; Fig. S4d). By the end of training sessions, we observed a similar pattern, with random and serial-oriented pattern (44% and 35% respectively; Fig. S4d). On the other hand, CI-R females were characterized by largely serial and spatial-oriented strategy (65% and 25% respectively; Fig. S4g). At the end of the training session, CI-R females acquired a combination of mostly serial (43%) and spatial (36%) strategy (Fig. S4g). Instead, CTL females applied mostly serial and random-oriented strategy (60% and 29% respectively) during day 1, changing to serial and spatial-oriented strategy by the end of the training period (69% and 29% respectively; Fig. S4a). In contrast, CI male degus, during day 1 of training, altered their search strategy from a combination of mostly random (52%) and serial (35%) strategy to a similar pattern with serial-random strategy as the most common (42 and 42% respectively; Fig. S4f) by the end of training sessions. Whereas, CI-R males were characterized by serial and random-oriented strategy (58% and 29% respectively), changing to a combination of serial and spatial-oriented strategy (47% and 39% respectively) at the end of the training period (Fig. S4h). Compared to CTL males, who applied the 3 strategies in a similar way during day 1 (serial: 38%; spatial: 31% and random: 31%), changing to a more spatial and serial-oriented strategy at the end of the training period (60 and 32% respectively) (Fig. S4b).

Effects of long-term social isolation on synaptic activity properties in brain slices of degus

We compared the fEPSP slopes vs stimulus strength within females (Fig. S6a) or males (Fig. S6b). We found no differences between treatments in females ($p = 0.622$) but we did it at males (two-way repeated measures ANOVA, Males: interaction: $F_{(24,72)} = 0.38$, $p = 0.995$; treatment: $F_{(3,72)} = 13.64$, $p < 0.001$; stimulus: $F_{(8,72)} = 10.49$, $p < 0.001$). Further analysis showed that CI-R's fEPSP slope is higher than CTL and CI, showing more response to the same stimulus. We also observed no-differences among treatments when we compared the FV amplitudes vs stimulus strength within females (Fig. S6a, $p = 0.052$) or males (Fig. S6b, $p = 0.057$), demonstrating that treatments do not affect the number of axons recruited, and so the excitability during basal synaptic transmission compared to CTL.

We found a significant positive correlation between FV amplitude and fEPSP slopes in females and males, in CTL, PI, CI and CI-R groups [Fig. S6c-d; CTL $female_{slope} = 0.028 \pm 0.001$, $R^2 = 0.990$, $F_{(1,7)} = 695.4$, $p < 0.01$; CTL $male_{slope} = 0.027 \pm 0.001$, $R^2 = 0.989$, $F_{(1,7)} = 660.5$, $p < 0.01$; PI $female_{slope} = 0.032 \pm 0.001$, $R^2 = 0.990$, $F_{(1,7)} = 688.7$, $p < 0.01$; PI $male_{slope} = 0.035 \pm 0.001$, $R^2 = 0.990$, $F_{(1,7)} = 663.2$, $p < 0.01$; CI $female_{slope} = 0.017 \pm 0.001$, $R^2 = 0.987$, $F_{(1,7)} = 519.7$, $p < 0.01$; CI $male_{slope} = 0.031 \pm 0.002$, $R^2 = 0.976$, $F_{(1,7)} = 280.2$, $p < 0.01$; CI-R $female_{slope} = 0.022 \pm 0.001$, $R^2 = 0.989$, $F_{(1,7)} = 622.9$, $p < 0.01$; CI-R $male_{slope} = 0.048 \pm 0.004$, $R^2 = 0.957$, $F_{(1,7)} = 155.9$, $p < 0.01$]. We also compared the linear regressions obtained from the correlation between FV amplitude and fEPSP slopes, within each sex. Within females (Fig. S6c) we found significant differences at slopes between females CTL and PI group (ANCOVA: $F_{(1,14)} = 5.09$, $p = 0.041$), at intercepts and slopes between CTL and CI group (ANCOVA intercept: $F_{(1,15)} = 25.54$, $p < 0.01$; slope: $F_{(1,14)} = 66.29$, $p < 0.01$), and significant at intercepts and slopes between CTL and CI-R group (ANCOVA intercept: $F_{(1,15)} = 17.10$, $p < 0.01$; slope: $F_{(1,14)} = 21.84$, $p < 0.01$), at intercept between PI and CI group (ANCOVA: $F_{(1,15)} = 27.15$, $p < 0.01$), at intercepts and slopes between PI and CI-R group (ANCOVA intercept: $F_{(1,15)} = 21.19$, $p < 0.01$; slope: $F_{(1,14)} = 46.91$, $p < 0.01$), at intercepts and slopes between CI and CI-R group (ANCOVA intercept: $F_{(1,15)} = 23.63$, $p < 0.01$; slope: $F_{(1,14)} = 13.28$, $p < 0.01$). These

data showed that, despite that PI is higher than CTL, the other groups are lower than CTL, demonstrating that females cannot accommodate synaptic transmission during stress treatments. Comparison within groups of males (Fig. S6d) obtain significant differences at intercepts and slopes between males CTL and PI group (ANCOVA intercept: $F_{(1,15)} = 37.00$, $p < 0.01$; slope: $F_{(1,14)} = 23.47$, $p < 0.01$), at intercepts and slopes between CTL and CI group (ANCOVA intercept: $F_{(1,15)} = 32.47$, $p < 0.01$; slope: $F_{(1,14)} = 4.96$, $p < 0.043$), significant at intercepts and slopes between CTL and CI-R group (ANCOVA intercept: $F_{(1,15)} = 65.04$, $p < 0.01$; slope: $F_{(1,14)} = 27.31$, $p < 0.01$), at intercept between PI and CI group (ANCOVA: $F_{(1,15)} = 4.69$, $p < 0.047$), at intercepts and slopes between PI and CI-R group (ANCOVA intercept: $F_{(1,15)} = 48.60$, $p < 0.01$; slope: $F_{(1,14)} = 9.96$, $p < 0.01$), at intercepts and slopes between CI and CI-R group (ANCOVA intercept: $F_{(1,15)} = 48.14$, $p < 0.01$; slope: $F_{(1,14)} = 10.05$, $p < 0.01$). In the case of males, every regression is above CTL, showing that males can efficiently modulate the basal transmission to stress treatments.

Within the LTP protocol, the comparison of the last 15 min within groups of females (Fig. S6e) generates non-significant differences between females ($p = 0.246$). However, comparison of the last 15 min within groups of males (Fig. S6f) generates significant differences (two-way repeated measures ANOVA: interaction: $F_{(30,77)} = 0.02$, $p = 1.00$, treatment $F_{(3,77)} = 35.62$, $p < 0.01$, time $F_{(10,77)} = 0.02$, $p = 1.00$). Further analysis indicates that CI-R is significantly lower than CTL (Bonferroni post-hoc test, CTL vs CI-R $p = 0.034$).

Canonical Wnt signaling-related proteins in hypothalamus, hippocampus, and prefrontal cortex

The effects of long-term chronic social isolation on the expression levels of total GSK3β in the hypothalamus of female degus indicated a significant effect of stress treatment [$F_{(3,8)} = 5.37$, $p = 0.02$], where CI females had higher values compared to CI-R (Fig. S7a). Instead, no changes were observed in males ($p = 0.20$; Fig. S7b), suggesting that re-socialization posterior to chronic SIS is able to decrease GSK3β protein amounts in female degus, which could be modulating its cellular effects indirectly. For the pY216-GSK3β levels we no found a significant effect of stress treatment in both female ($p = 0.26$; Fig. S7a) and male groups ($p = 0.16$; Fig. S7b). For the pS9-GSK3β levels in the hypothalamus of female degus, we found a significant effect of stress treatment [$F_{(3,8)} = 21.88$, $p < 0.01$]. Comparisons between groups indicated that CI female degus have higher values compared with CTL and CI-R groups. Instead, no changes were observed in males ($p = 0.08$; Fig. S7a-b).

The expression of total GSK3β in the hippocampus of female degus was not altered by the stress treatment ($p = 0.46$; Fig. S7c). Instead, male degus showed a significant effect of the stress treatment [$F_{(3,8)} = 10.20$, $p < 0.01$], where CI group showed lower values compared to PI, and CI-R males presented lower values compared to CTL and PI groups (Fig. S7d). This result, suggested that social isolation stress could be regulating GSK3β protein expression dynamic and its cellular effects in male degus. Moreover, the levels of pY216-GSK3β in female degus were no changed across stress treatment ($p = 0.11$; S7c), whereas, male degus showed a significant effect of stress treatment [$F_{(3,8)} = 5.25$, $p = 0.02$], with CI-R males showed a dramatic decrease of this protein compared with the other groups (Fig. S7d). On the other hand, expression of pS9-GSK3β in the hippocampus showed no changes across stress treatments along female and male degus ($p = 0.13$ and $p = 0.25$, respectively; Fig. S7c-d).

For the expression of total GSK3β levels in the prefrontal cortex of female and male degus, the one-way ANOVA revealed a significant effect of stress treatment [$F_{(3,8)}$=9.91, $p < 0.01$ and

$F_{(3,8)}$=4.23, $p = 0.04$, respectively]. Comparisons between groups indicated that both female and male degus under CI-R treatment have higher values of total GSK3β than the CI group (Fig. S7e-f). Contrary to the hypothalamus, these results suggest that resocialization increased GSK3β protein amount, in a sex-independent manner, which could be differently regulating its cellular effects. For the expression of pY216-GSK3β in both female and male degus, we observed no changes across stress treatments along female and male degus ($p = 0.38$ and $p = 0.12$, respectively; Fig. S7e-f). Similarly, for the pS9-GSK3β we observed no changes across stress treatments along female and male degus ($p = 0.09$ and $p = 0.17$, respectively; Fig. S7e-f).

Table 1.

The p-value for comparisons among stress treatments for each time (days) during the training phase. LTEH: latency to find the escape hole; MRE: reference memory errors; WRE: working memory errors. Values in boldface type indicate statistically significant differences at $p < 0.05$.

	**Day**						
♀	1	2	3	4	5	6	7
**LTEH**	0.36	0.47	0.44	0.23	**0.02**	0.23	0.24
♂	1	2	3	4	5	6	7
**LTEH**	**0.03**	**<0.01**	**<0.01**	**<0.01**	**0.03**	**<0.01**	**<0.01**
♀	1	2	3	4	5	6	7
**MRE**	0.07	**<0.01**	**0.01**	**0.03**	**0.03**	**<0.01**	0.19
♂	1	2	3	4	5	6	7
**MRE**	0.81	0.29	**<0.01**	0.05	0.10	0.19	**<0.01**
♀	1	2	3	4	5	6	7
**WRE**	**0.04**	**<0.01**	**0.01**	**0.01**	**<0.01**	**<0.01**	0.39
♂	1	2	3	4	5	6	7
**WRE**	0.46	0.07	**<0.01**	0.05	0.44	0.28	**<0.01**

Figure legends

Fig. S1. Effect of long-term chronic social isolation stress in the exploration behaviour on the open field test: (a) time spent in the central zone of the arena (b) number of central crossings (b) average speed (c) total distance travelled. Control (CTL), Partial Isolation (PI), Chronic Isolation (CI), and Re-socialization (CI-R) treatment groups. The data were analysed statistically using two-way ANOVA followed by Fisher's LSD post hoc test. The effect of stress treatment, sex, and the interaction between the two factors are indicated in the top of the figure. Each symbol corresponds to data from a single sex-group, represented as the mean ± SEM (n = 13).

Fig. S2. Effect of long-term chronic social isolation stress in working memory on the NLR/NOR test (a) average interaction time with familiar object A and novel local object B during NLR task (b) average interaction time with familiar object A and novel object B during NOR task. Control (CTL), Partial Isolation (PI), Chronic Isolation (CI), and Re-socialization (CI-R) treatment groups. The data were analysed statistically using two-way ANOVA followed by Fisher's LSD post hoc test. The effect of stress treatment, sex, and the interaction between the two factors are indicated in the top of the figure. Each symbol corresponds to data from a single sex-group, represented as the mean ± SEM (n = 13).

Fig. S3. Long-term chronic social isolation stress causes a gender-dependent and temporal specific effect on Barnes maze test (a-b) learning curve of latency of the first visit to escape hole (c-d) learning curve of the reference memory errors (e-f) learning curve of working memory errors. Control (CTL), Partial Isolation (PI), Chronic Isolation (CI), and Re-socialization (CI-R) treatment groups. Results are expressed as mean ± SEM (n = 13). MSE = mean standard error.

Fig. S4. The navigation search strategy utilized during training sessions causes a gender-dependent and temporal specific effects on the Barnes maze test. S: serial strategy; E: spatial strategy; R: random strategy. (a-b) Control (CTL) (c-d) Partial Isolation (PI) (e-f) Chronic Isolation (CI) (g-h) Re-socialized (CI-R). The 'random strategy' was defined as searches with no systematic search

pattern or when searches of scape hole were interrupted by central crosses. The ‘serial searches’ were defined as searches of consecutive holes around the maze, and ‘spatial searches’ were defined as searches following a direct path to the escape hole.

Fig. S5. Effect of long-term chronic social isolation stress in the exploration behaviour on the Barnes maze test: (a) average speed (b) total distance travelled. Control (CTL), Partial Isolation (PI), Chronic Isolation (CI), and Re-socialization (CI-R) treatment groups. The data were analysed statistically using two-way ANOVA followed by Fisher’s LSD post hoc test. The effect of stress treatment, sex, and the interaction between the two factors are indicated in the top of the figure. Each symbol corresponds to data from a single sex-group, represented as the mean ± SEM (n = 13).

Fig. S6. Electrophysiological measurements: comparison within groups. (a-b) field excitatory postsynaptic potentials (fEPSPs) slopes (upper plots) and fiber volley (FV) amplitudes (lower plots) per stimulus strength at each experimental condition, in females (a) and males (b). (c-d) Correlation and regression plot between FV amplitude and fEPSP slopes of each experimental group, in females (c) and males (d). (e-f) Comparison of long-term potentiation (LTP) induction on each experimental group in females (e) and males (f). Analyses were performed by two-way repeated measures ANOVA and Bonferroni post hoc test, *$p < 0.05$.

Fig. S7. Biochemical analysis of hypothalamic, hippocampal, and prefrontal cortex (PFC) canonical Wnt signalling-related proteins. Densitometric analysis of (a-b) hypothalamic (c-d) hippocampal and (e-f) PFC of total GSK3β, pY216-GSK3β, and pS9-GSK3β of female degus and male degus. Data were analysed statistically using one-way ANOVAs, with the p-value indicated at the top of each figure. Different letters above bars show statistical differences between the same protein across stress treatments (Fisher’s LSD *post hoc* test). Results are expressed as mean ± SE (n = 3). a.u: arbitrary units. Control (CTL), Partial Isolation (PI), Chronic Isolation (CI), and Re-socialization (CI-R) treatment groups.

Fig. S8. Schematic summary of dynamic of synaptic and canonical Wnt signalling-related proteins in (a) hypothalamus of female and male (b) hippocampus of female and male (c) prefrontal cortex of female and male degus. Control (CTL), Partial Isolation (PI), Chronic Isolation (CI), and Re-socialization (CI-R) treatment groups.

Figure S1

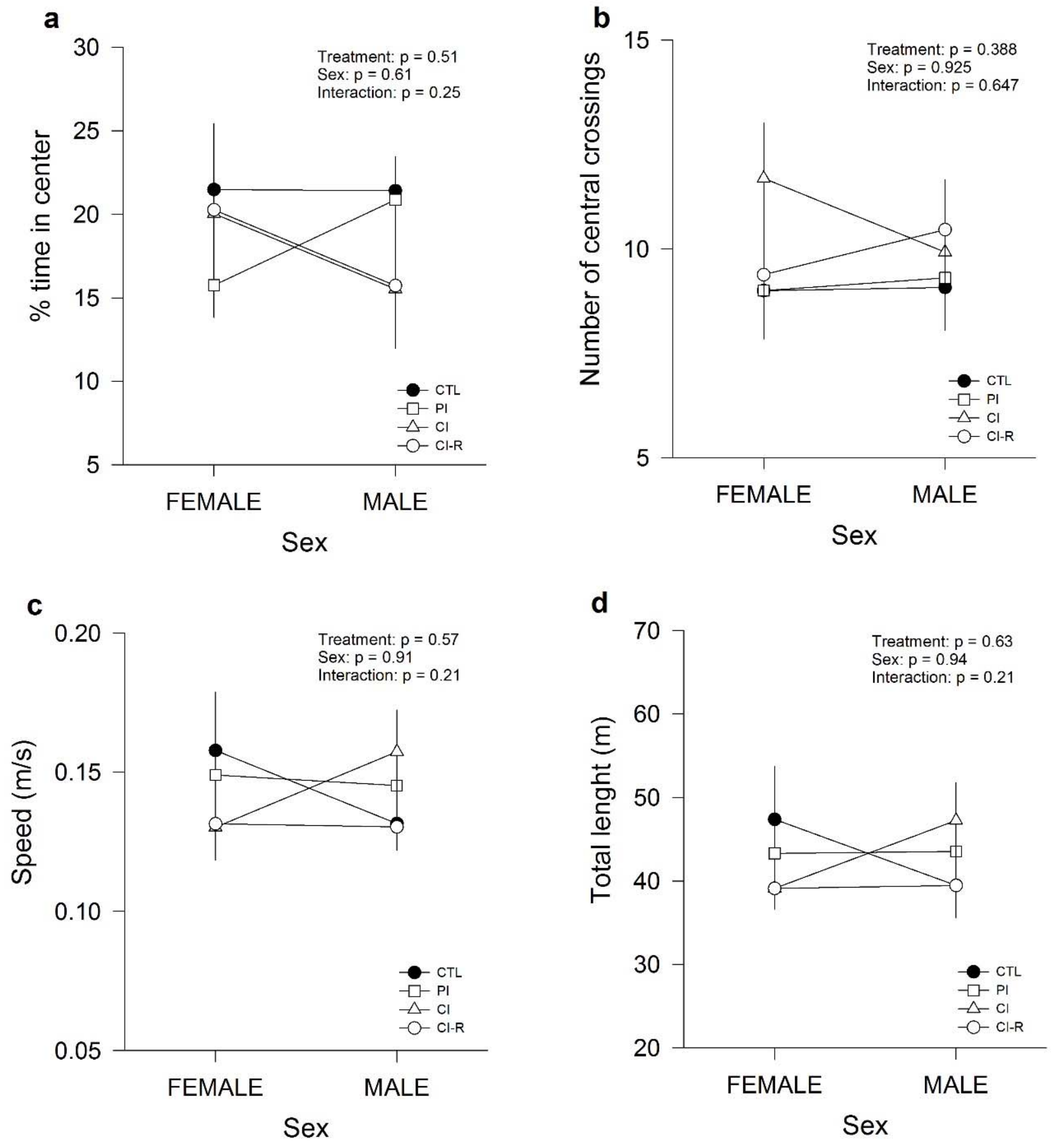
a
% time in center
30
25
20
15
10
5
Treatment: p = 0.51
Sex: p = 0.61
Interaction: p = 0.25
CTL
PI
CI
CI-R
FEMALE
MALE
Sex
b
Number of central crossings
15
10
5
Treatment: p = 0.388
Sex: p = 0.925
Interaction: p = 0.647
CTL
PI
CI
CI-R
FEMALE
MALE
Sex
c
Speed (m/s)
0.20
0.15
0.10
0.05
Treatment: p = 0.57
Sex: p = 0.91
Interaction: p = 0.21
CTL
PI
CI
CI-R
FEMALE
MALE
Sex
d
Total lenght (m)
70
60
50
40
30
20
Treatment: p = 0.63
Sex: p = 0.94
Interaction: p = 0.21
CTL
PI
CI
CI-R
FEMALE
MALE
Sex

Figure S2

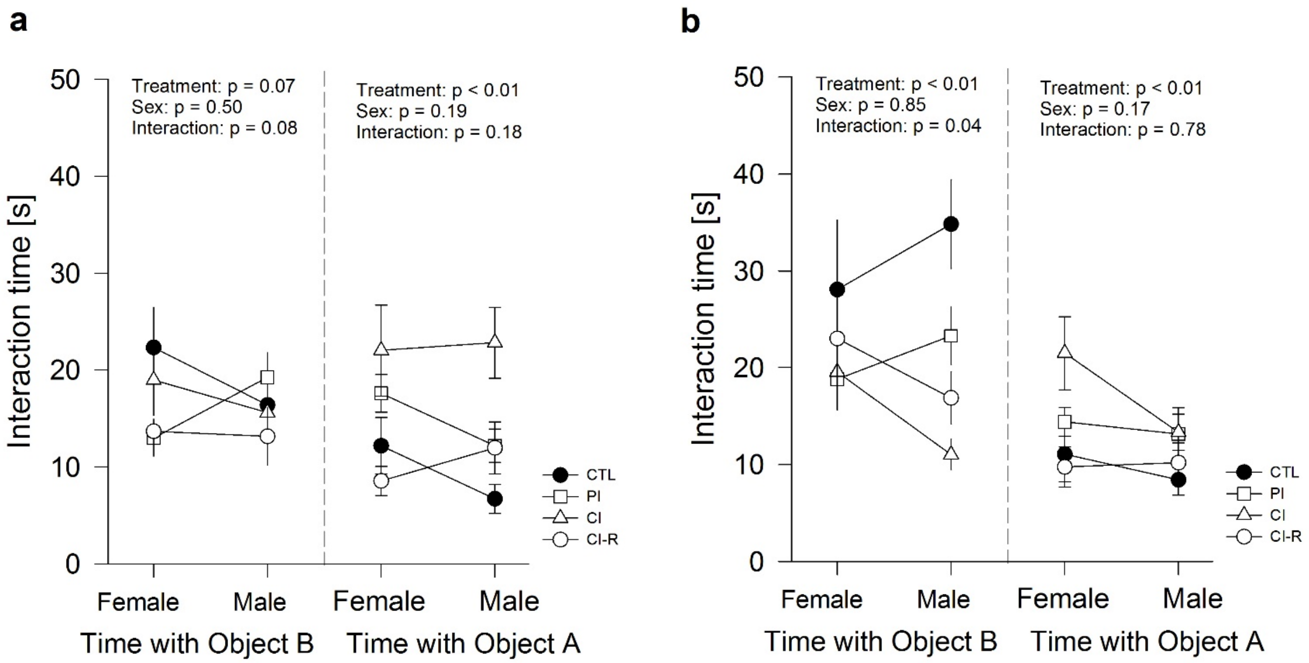

a
Treatment: p = 0.07
Sex: p = 0.50
Interaction: p = 0.08
Treatment: p < 0.01
Sex: p = 0.19
Interaction: p = 0.18
Interaction time [s]
Female
Male
Female
Male
Time with Object B
Time with Object A
CTL
PI
CI
CI-R
b
Treatment: p < 0.01
Sex: p = 0.85
Interaction: p = 0.04
Treatment: p < 0.01
Sex: p = 0.17
Interaction: p = 0.78
Interaction time [s]
Female
Male
Female
Male
Time with Object B
Time with Object A
CTL
PI
CI
CI-R

Figure S3

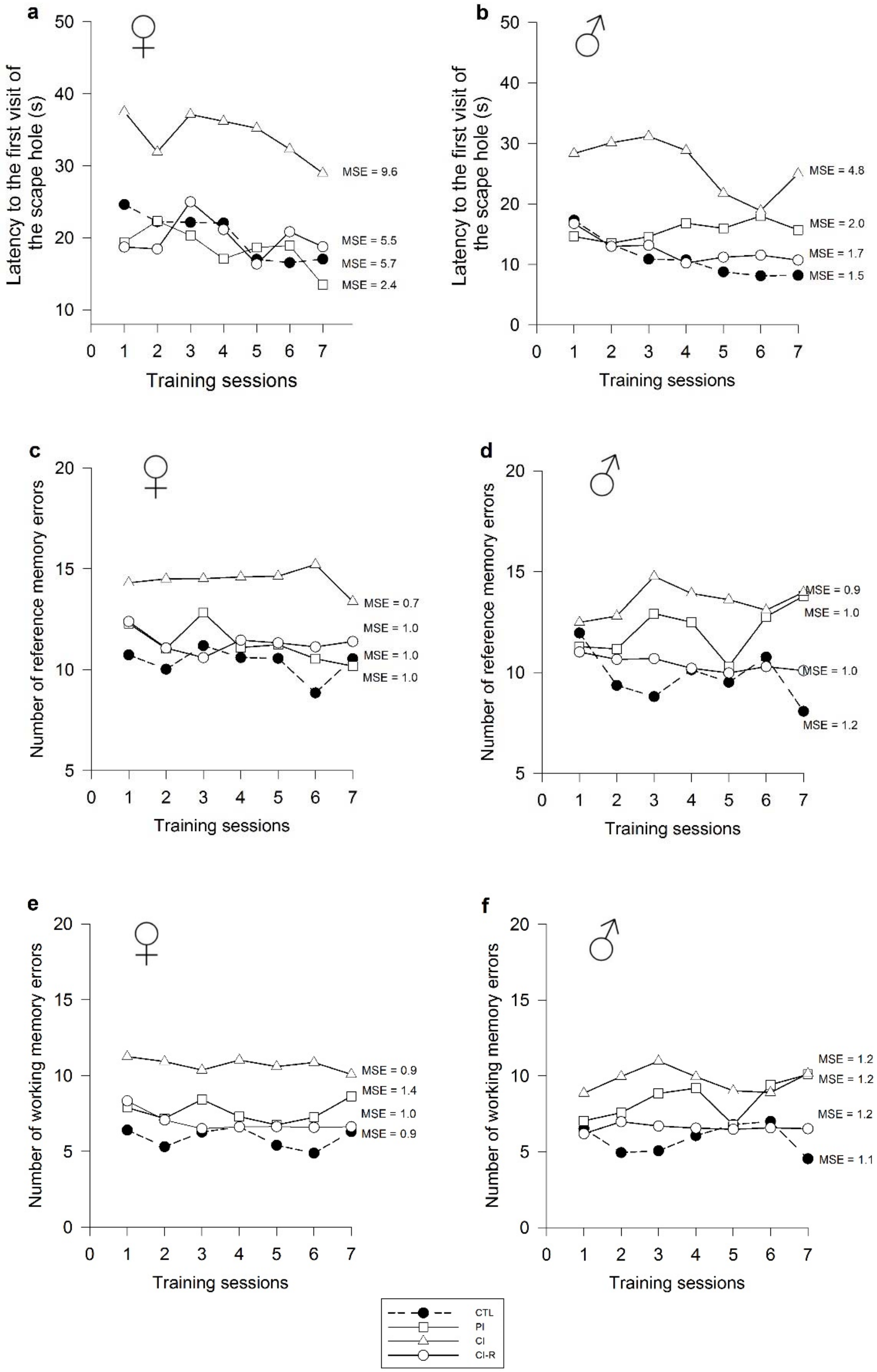
a
b
c
d
e
f
Latency to the first visit of the scape hole (s)
Number of reference memory errors
Number of working memory errors
Training sessions
MSE = 9.6
MSE = 5.5
MSE = 5.7
MSE = 2.4
MSE = 4.8
MSE = 2.0
MSE = 1.7
MSE = 1.5
MSE = 0.7
MSE = 1.0
MSE = 1.0
MSE = 1.0
MSE = 0.9
MSE = 1.0
MSE = 1.0
MSE = 1.2
MSE = 0.9
MSE = 1.4
MSE = 1.0
MSE = 0.9
MSE = 1.2
MSE = 1.2
MSE = 1.2
MSE = 1.1
CTL
PI
CI
CI-R

Figure S4

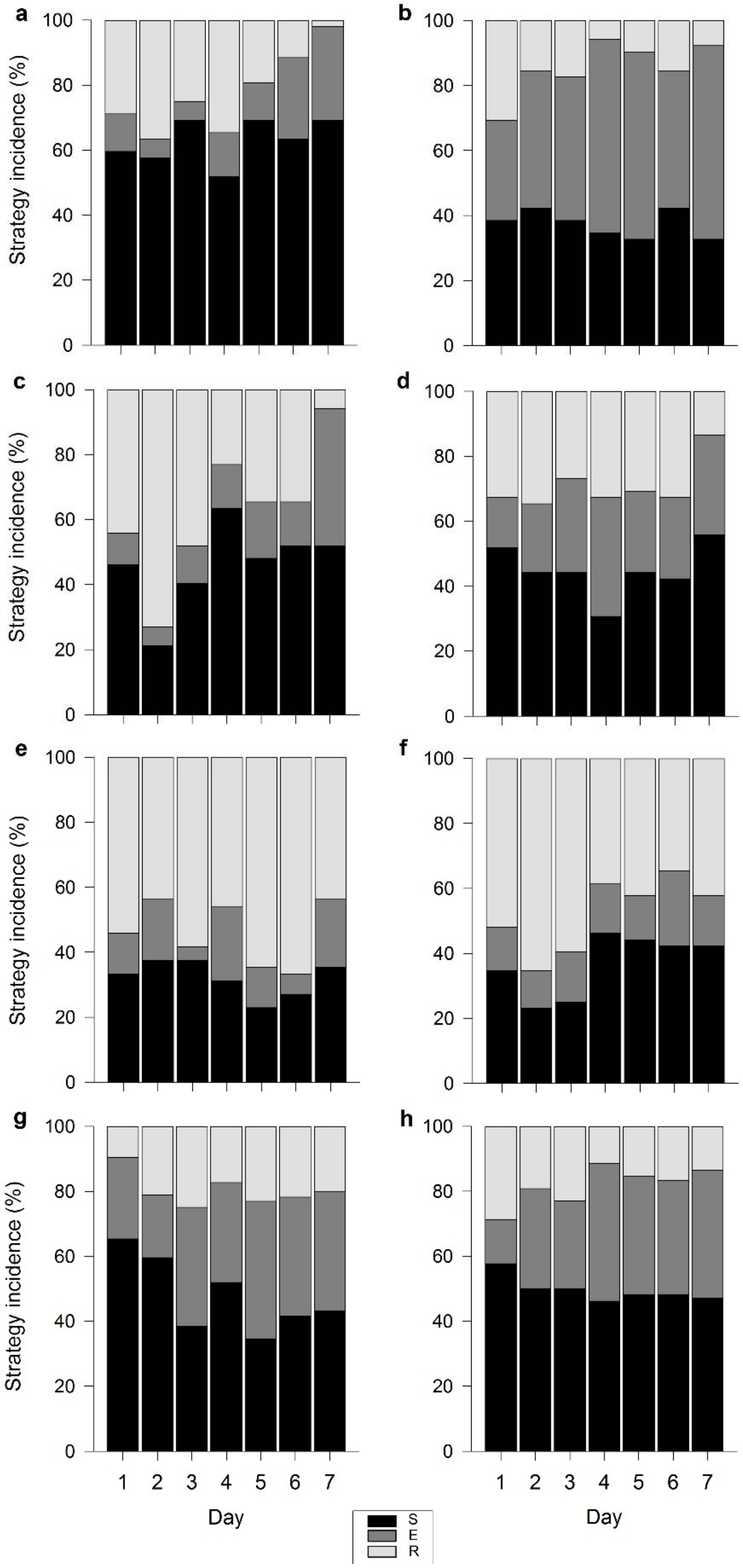

a
b
c
d
e
f
g
h
Strategy incidence (%)
0
20
40
60
80
100
1
2
3
4
5
6
7
Day
S
E
R

Figure S5

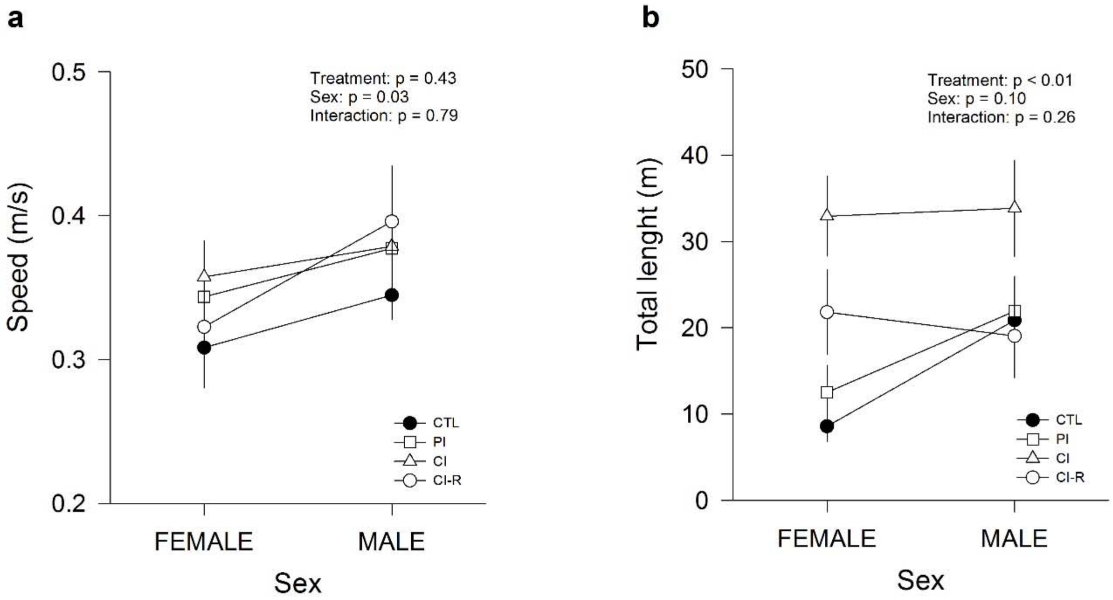
a
0.5
0.4
0.3
0.2
Speed (m/s)
Treatment: p = 0.43
Sex: p = 0.03
Interaction: p = 0.79
CTL
PI
CI
CI-R
FEMALE
MALE
Sex
b
50
40
30
20
10
0
Total lenght (m)
Treatment: p < 0.01
Sex: p = 0.10
Interaction: p = 0.26
CTL
PI
CI
CI-R
FEMALE
MALE
Sex

Figure S6

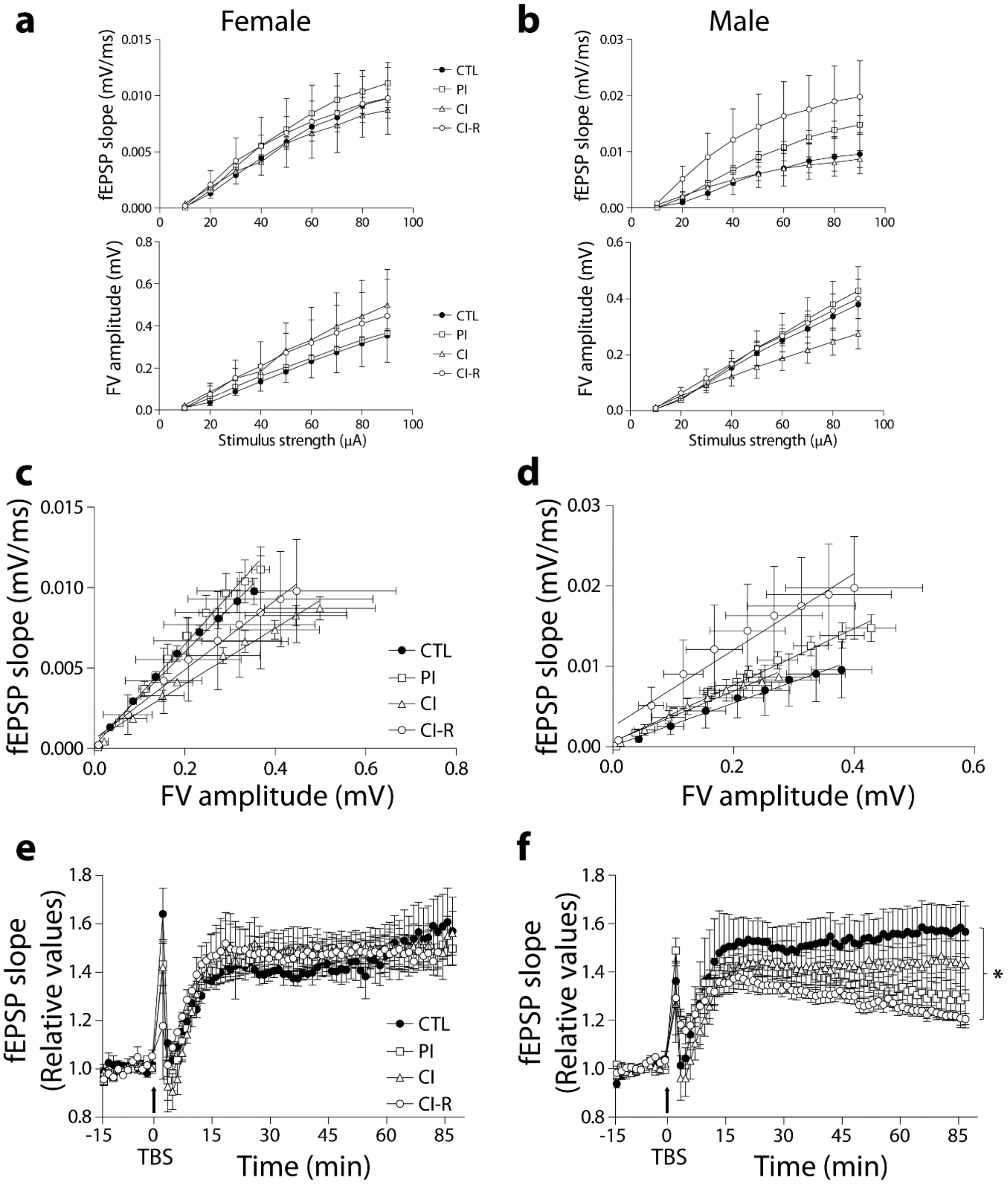

a
Female
fEPSP slope (mV/ms)
CTL
PI
CI
CI-R
FV amplitude (mV)
Stimulus strength (μA)
b
Male
fEPSP slope (mV/ms)
FV amplitude (mV)
Stimulus strength (μA)
c
fEPSP slope (mV/ms)
FV amplitude (mV)
CTL
PI
CI
CI-R
d
fEPSP slope (mV/ms)
FV amplitude (mV)
e
fEPSP slope (Relative values)
TBS
Time (min)
CTL
PI
CI
CI-R
f
fEPSP slope (Relative values)
TBS
Time (min)
*

Figure S7

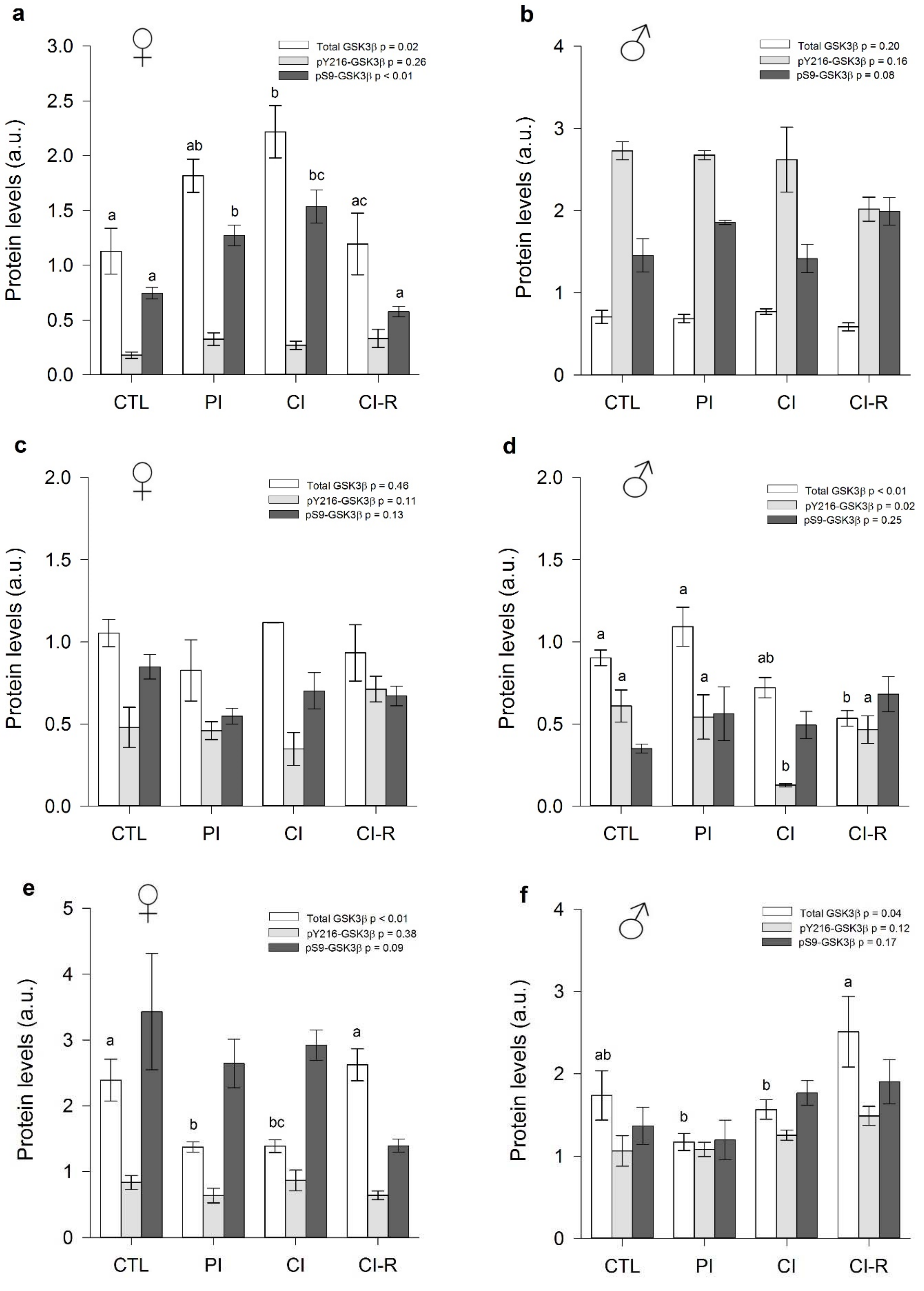
a
♀
Total GSK3β p = 0.02
pY216-GSK3β p = 0.26
pS9-GSK3β p < 0.01
Protein levels (a.u.)
CTL
PI
CI
CI-R
b
♂
Total GSK3β p = 0.20
pY216-GSK3β p = 0.16
pS9-GSK3β p = 0.08
Protein levels (a.u.)
CTL
PI
CI
CI-R
c
♀
Total GSK3β p = 0.46
pY216-GSK3β p = 0.11
pS9-GSK3β p = 0.13
Protein levels (a.u.)
CTL
PI
CI
CI-R
d
♂
Total GSK3β p < 0.01
pY216-GSK3β p = 0.02
pS9-GSK3β p = 0.25
Protein levels (a.u.)
CTL
PI
CI
CI-R
e
♀
Total GSK3β p < 0.01
pY216-GSK3β p = 0.38
pS9-GSK3β p = 0.09
Protein levels (a.u.)
CTL
PI
CI
CI-R
f
♂
Total GSK3β p = 0.04
pY216-GSK3β p = 0.12
pS9-GSK3β p = 0.17
Protein levels (a.u.)
CTL
PI
CI
CI-R

Figure S8

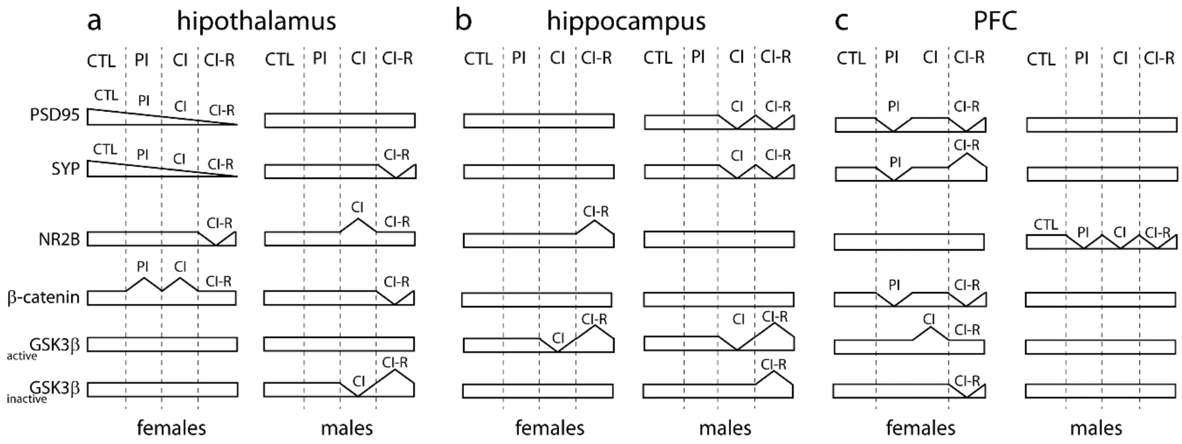
a
hipothalamus
b
hippocampus
c
PFC
CTL
PI
CI
CI-R
PSD95
SYP
NR2B
β-catenin
active GSK3β
inactive GSK3β
females
males
