# Supplementary material for: Effects of long-lasting social isolation and re-socialization on cognitive performance and brain activity: a longitudinal study in Octodon degus
Source: Sci Rep. 2020 Oct 27;10:18315. doi: 10.1038/s41598-020-75026-4 (PMC7591540; doi:10.1038/s41598-020-75026-4)

Effects of long-lasting social isolation and re-socialization on cognitive performance and brain activity: a longitudinal study in *Octodon degus*

Daniela S. Rivera, Carolina B. Lindsay, Carolina A. Oliva, Juan Francisco Codocedo, Francisco Bozinovic and Nibaldo C. Inestrosa

Here we show the original images of the western blots used and analyzed in our study. Each image is labeled in the upper-side: Brain region, animal's sex and targeted protein. Each well corresponds to an individual animal sample, and three animals were used per treatment: Well 1-3=CTL, Well 4-6=PI, Well 7-9=CI and Well 10-12=CI-R.

Methods: In our experimental design, once we have transfered the gel to the PDVF membrane, we proceed to separate the membrane in two pieces by an horizontal cut through the 70kDa marker to perform a better and efficient use of the samples and antibodies supplies (except when the targeted protein weight is about 70kDa). Thus, all blot images show the upper (for >70 kDa proteins) or the bottom (for <70 kDa proteins) part of the transfered membrane.
Images were taken using a UPV Chemidoc-it Imaging System from Thermo Fisher Scientific.

(*) In these cases, the image showed here corresponds to a crop from the original western blot image, taken using a film-depending imaging system. Due to the COVID19-quarentine, we are not able to access to the original film or the computarized-whole scan. We apologize for this.

Hypothalamus Females NR2B

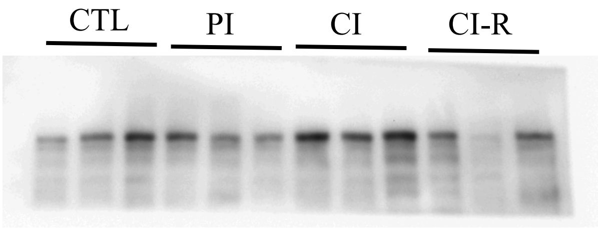

Hypothalamus Males NR2B

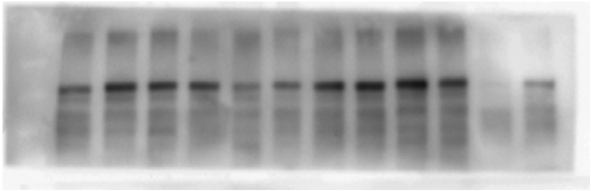

Hypothalamus Females PSD95*

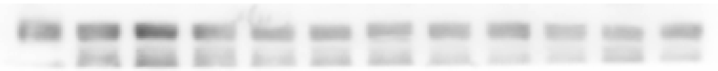

Hypothalamus Males PSD95

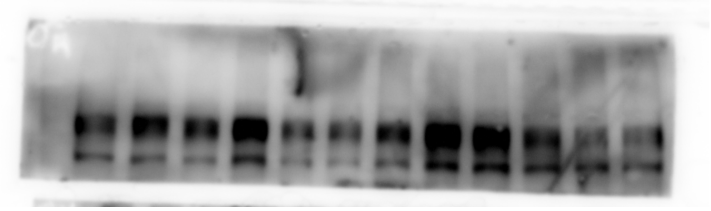

Hypothalamus Females SYP*

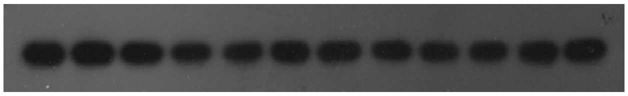

Hypothalamus Males SYP*

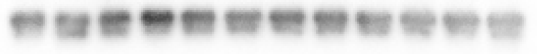

Hypothalamus Females Total GSK3β

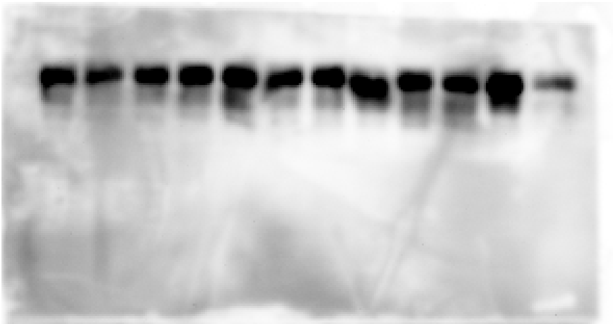

Hypothalamus Males Total GSK3β

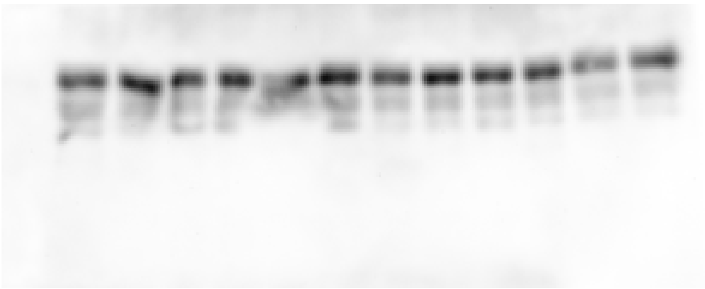

Hypothalamus Females Total β-catenin

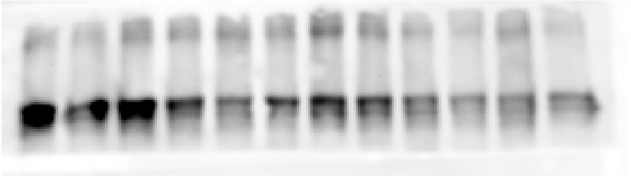

Hypothalamus Males Total β-catenin

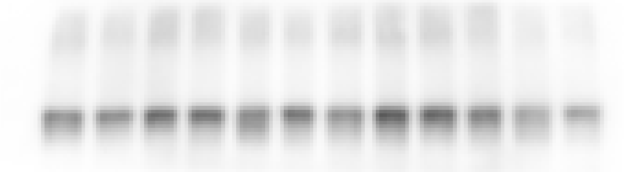

Hypothalamus Females β-actin*

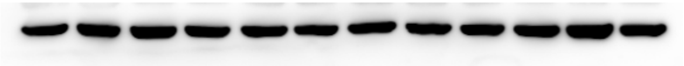

Hypothalamus Males β-actin*

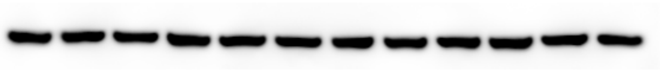

Hypothalamus Females pY216-GSK3β

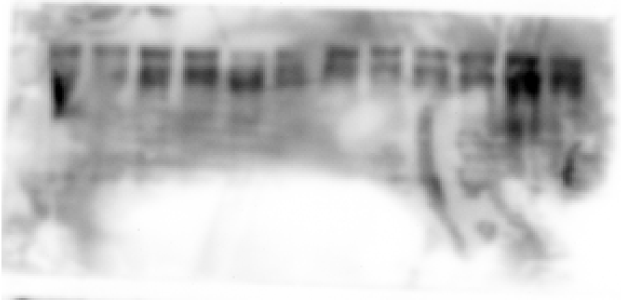

Hypothalamus Males pY216-GSK3β

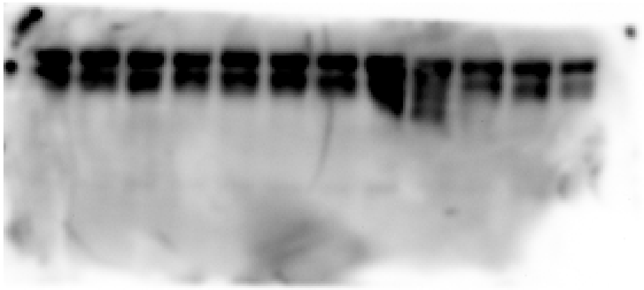

Hypothalamus Females pS9-GSK3β

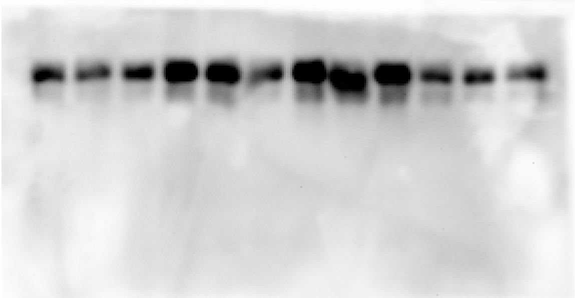

Hypothalamus Males pS9-GSK3β

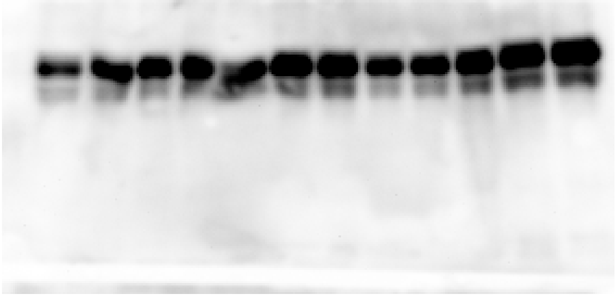

Hyppocampus Females actin

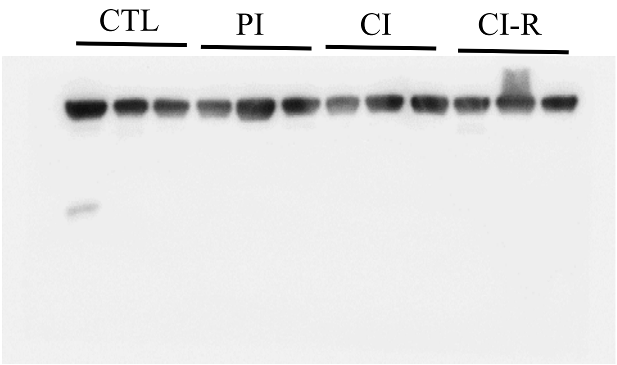

Hyppocampus Males actin

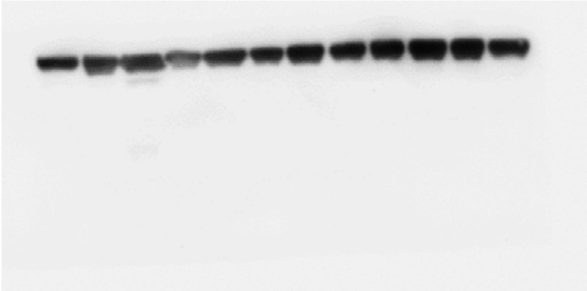

Hyppocampus Females PSD95

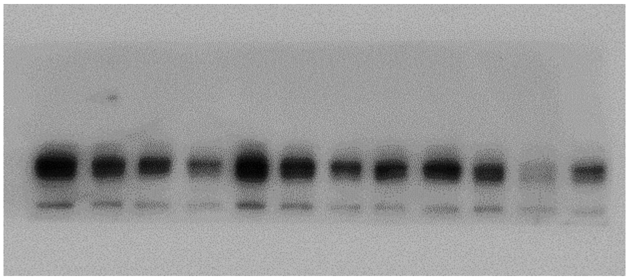

Hyppocampus Males PSD95

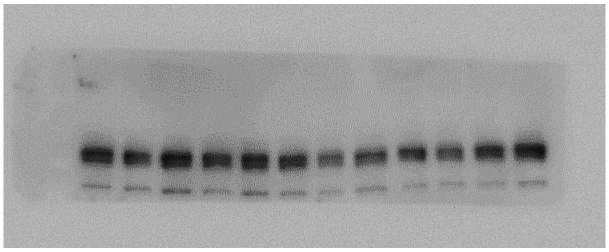

Hyppocampus Females SYP

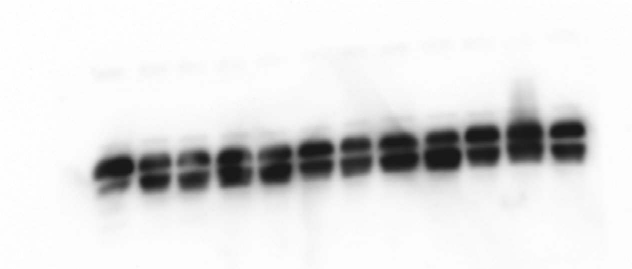

Hyppocampus Males SYP

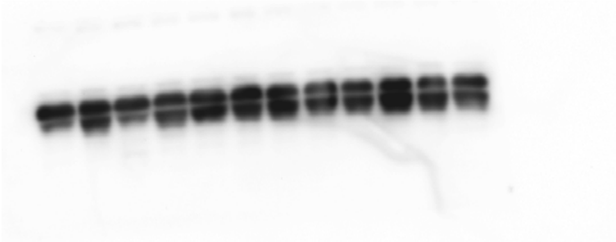

Hyppocampus Females NR2B

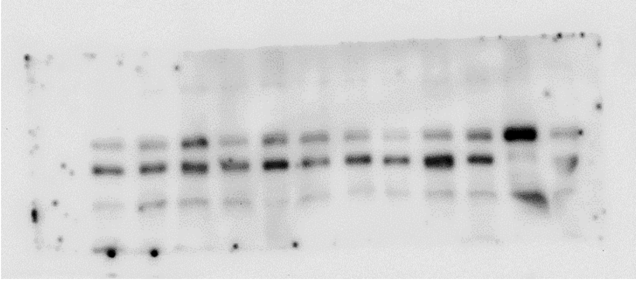

Hyppocampus Males NR2B male

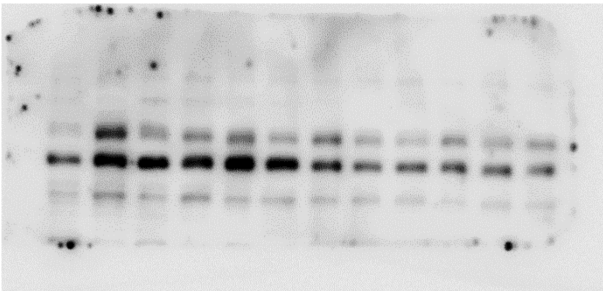

Hyppocampus Females Total β-catenin

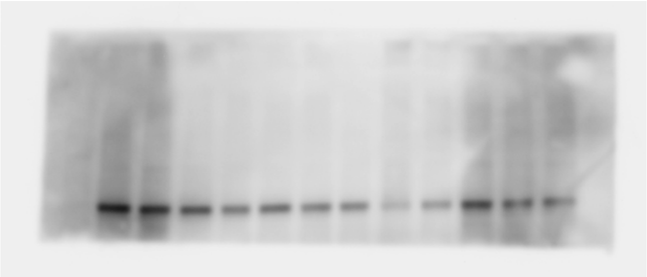

Hyppocampus Males Total β-catenin

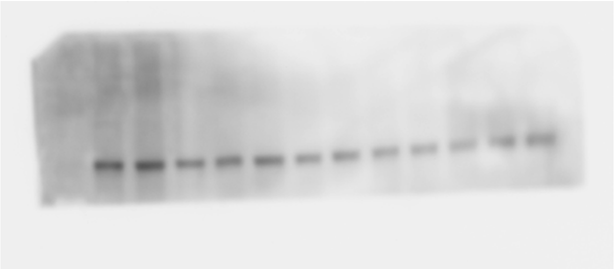

Hyppocampus Females pY216-GSK3β

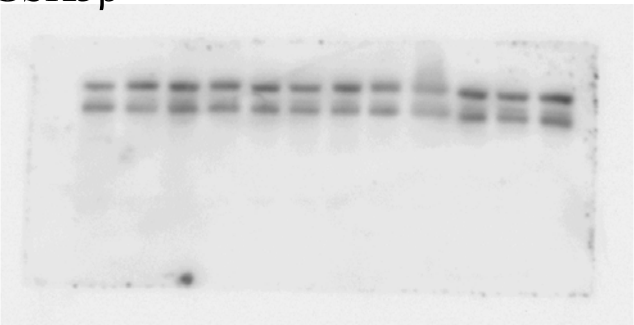

Hyppocampus Males pY216-GSK3β

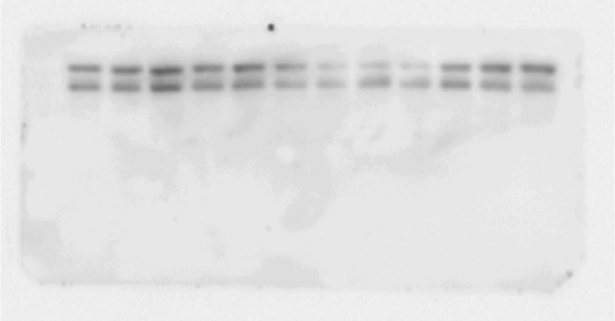

Hyppocampus Females pS9-GSK3β

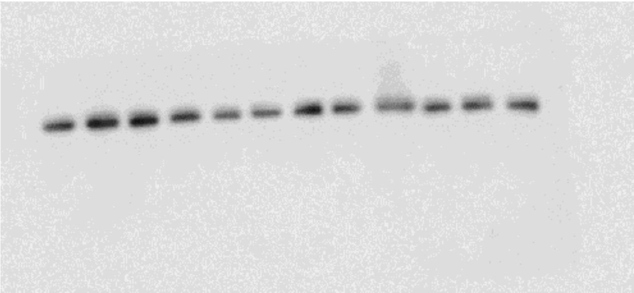

Hyppocampus Males pS9-GSK3β

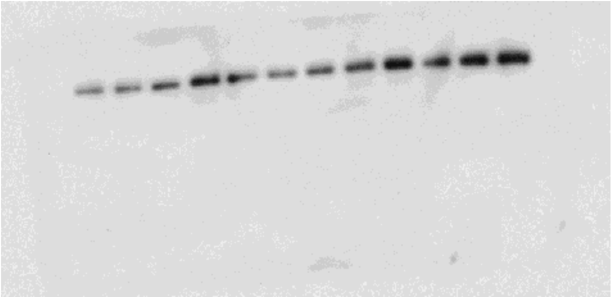

Hyppocampus Females Total GSK3β

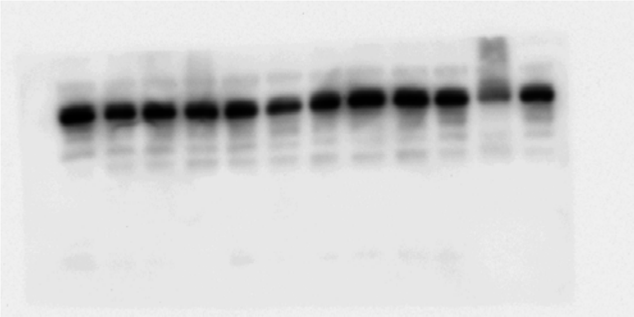

Hyppocampus Males Total GSK3β

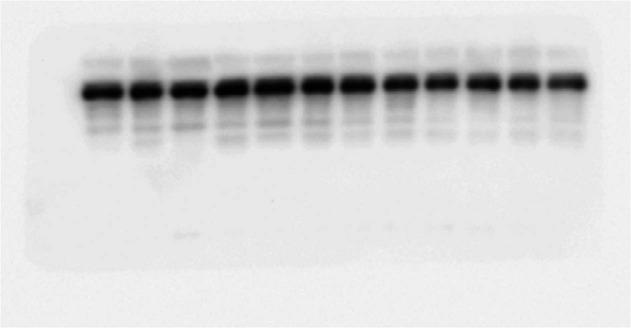

Prefrontal Cortex Female GAPDH

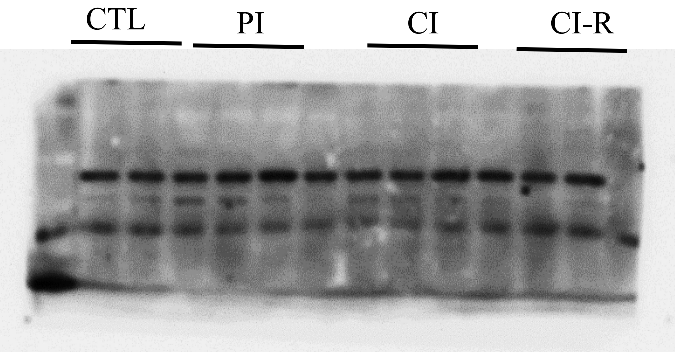

Prefrontal Cortex Male GAPDH

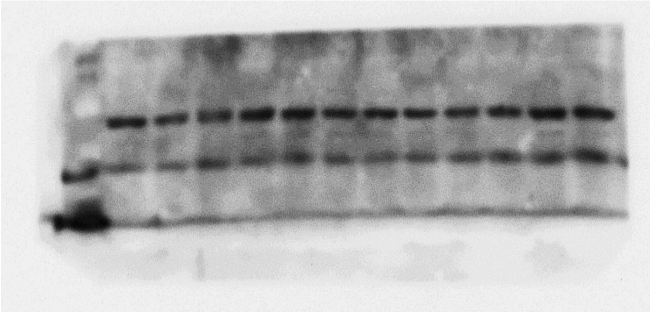

Prefrontal Cortex Female PSD95

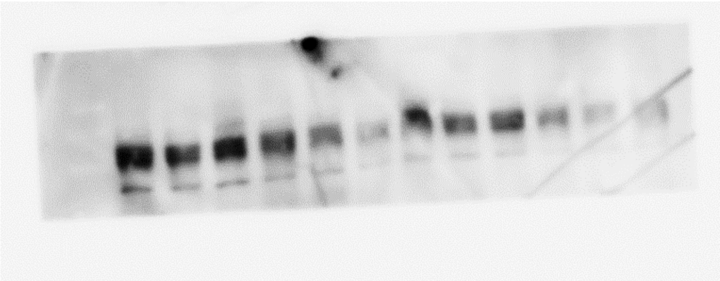

Prefrontal Cortex Male PSD95

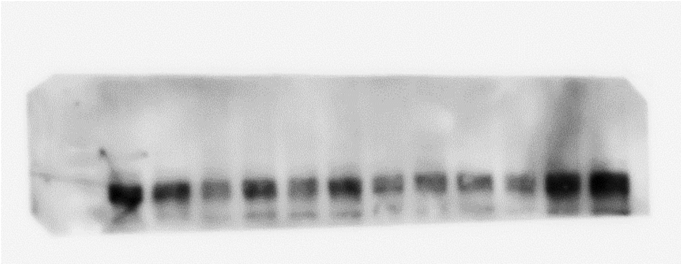

Prefrontal Cortex Female SYP

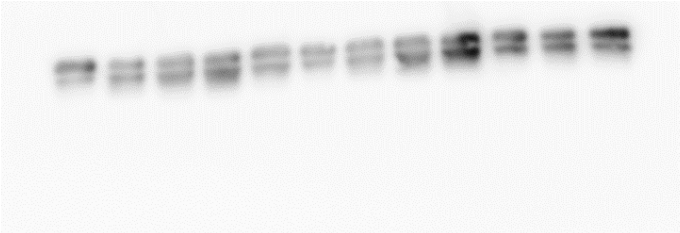

Prefrontal Cortex Male SYP

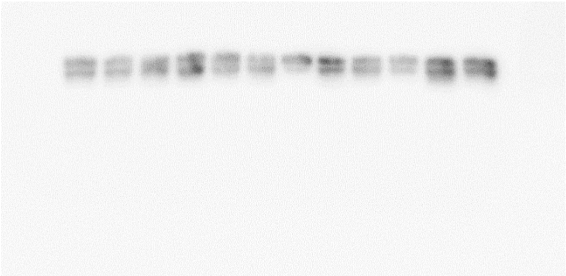

Prefrontal Cortex Female NR2B

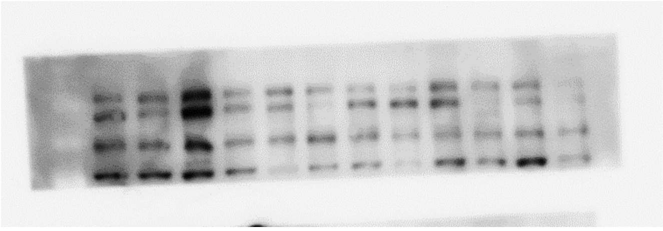

Prefrontal Cortex Male NR2B

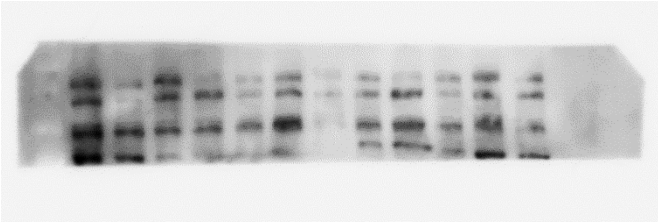

Prefrontal Cortex Female Total β-catenin

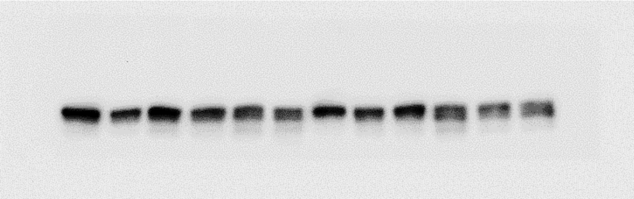

Prefrontal Cortex Female pY216-GSK3β

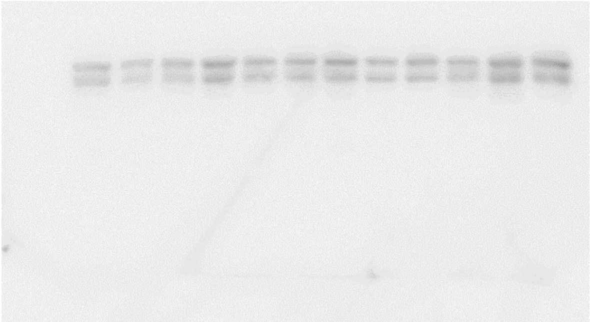

Prefrontal Cortex Female pS9-GSK3β

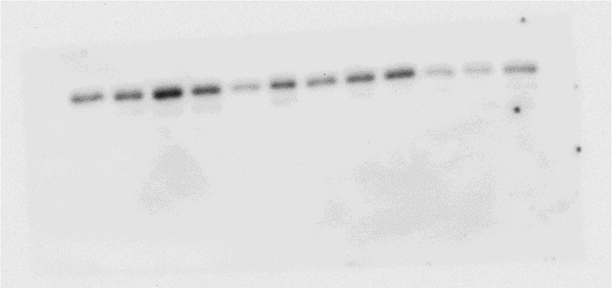

Prefrontal Cortex Female Total GSK3β

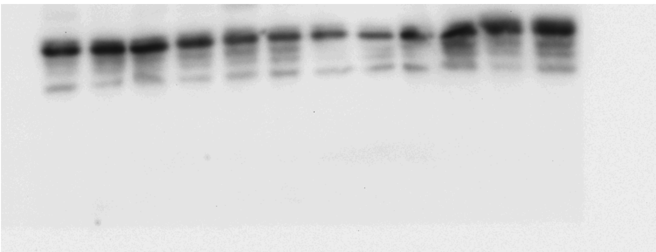

Prefrontal Cortex Male Total β-catenin

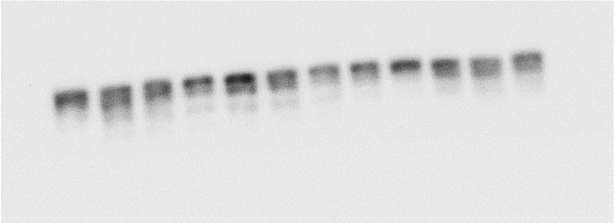

Prefrontal Cortex Male pY216-GSK3β

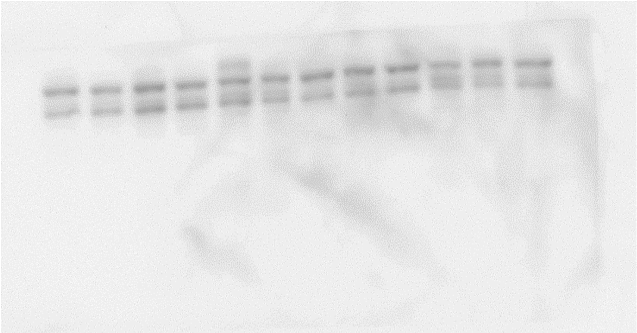

Prefrontal Cortex Male pS9-GSK3β

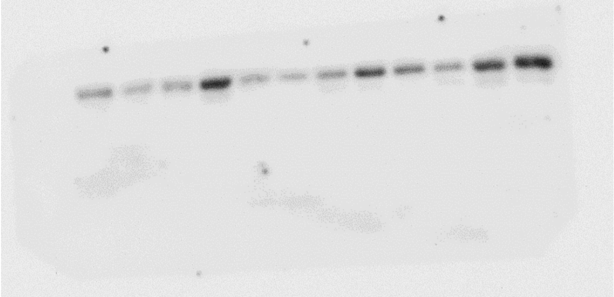

Prefrontal Cortex Male Total GSK3β

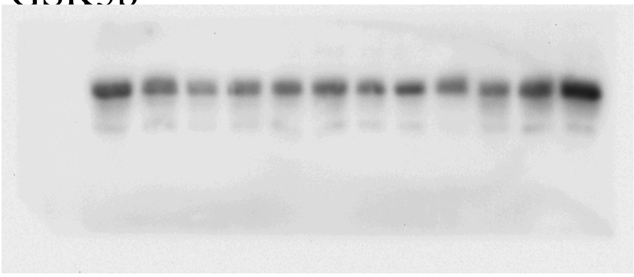

Supplement: Supplementary file 2 — Supplementary information 2. [file 41598_2020_75026_MOESM2_ESM.pdf]
